# Chromosome‐scale assembly of the genome of *Salix* *dunnii* reveals a male‐heterogametic sex determination system on chromosome 7

**DOI:** 10.1111/1755-0998.13362

**Published:** 2021-03-16

**Authors:** Li He, Kai‐Hua Jia, Ren‐Gang Zhang, Yuan Wang, Tian‐Le Shi, Zhi‐Chao Li, Si‐Wen Zeng, Xin‐Jie Cai, Natascha Dorothea Wagner, Elvira Hörandl, Aline Muyle, Ke Yang, Deborah Charlesworth, Jian‐Feng Mao

**Affiliations:** ^1^ Beijing Advanced Innovation Center for Tree Breeding by Molecular Design National Engineering Laboratory for Tree Breeding Key Laboratory of Genetics and Breeding in Forest Trees and Ornamental Plants Ministry of Education College of Biological Sciences and Technology Beijing Forestry University Beijing China; ^2^ College of Forestry Fujian Agriculture and Forestry University Fuzhou China; ^3^ Ori (Shandong) Gene Science and Technology Co., Ltd Weifang China; ^4^ Department of Systematics, Biodiversity and Evolution of Plants (with Herbarium) University of Goettingen Göttingen Germany; ^5^ Department of Ecology and Evolutionary Biology University of California Irvine Irvine CA USA; ^6^ Institute of Evolutionary Biology School of Biological Sciences University of Edinburgh Edinburgh UK

**Keywords:** gene expression, genome‐wide association, long terminal repeat‐retrotransposons, sex‐linked region, XX/XY

## Abstract

Sex determination systems in plants can involve either female or male heterogamety (ZW or XY, respectively). Here we used Illumina short reads, Oxford Nanopore Technologies (ONT) long reads and Hi‐C reads to assemble the first chromosome‐scale genome of a female willow tree (*Salix dunnii*), and to predict genes using transcriptome sequences and available databases. The final genome sequence of 328 Mb in total was assembled in 29 scaffolds, and includes 31,501 predicted genes. Analyses of short‐read sequence data that included female and male plants suggested a male heterogametic sex‐determining factor on chromosome 7, implying that, unlike the female heterogamety of most species in the genus *Salix*, male heterogamety evolved in the subgenus *Salix*. The *S. dunnii* sex‐linked region occupies about 3.21 Mb of chromosome 7 in females (representing its position in the X chromosome), probably within a pericentromeric region. Our data suggest that this region is enriched for transposable element insertions, and about one‐third of its 124 protein‐coding genes were gained via duplications from other genome regions. We detect purifying selection on the genes that were ancestrally present in the region, though some have been lost. Transcriptome data from female and male individuals show more male‐ than female‐biased genes in catkin and leaf tissues, and indicate enrichment for male‐biased genes in the pseudo‐autosomal regions. Our study provides valuable genomic resources for further studies of sex‐determining regions in the family Salicaceae, and sex chromosome evolution.

## 1 INTRODUCTION

Dioecious plants are found in ~5%–6% of flowering plant species (Charlesworth, 1985; Renner, 2014), and genetic sex determination systems have evolved repeatedly among flowering plants, and independently in different lineages. Some species have pronounced morphological differences between their sex chromosomes (heteromorphism), while others have homomorphic sex chromosomes (reviewed by Ming et al., 2011; Westergaard, 1958). Among homomorphic systems, some are young, with only small divergence between Y‐ and X‐linked sequences (e.g., Veltsos et al., 2019). Recent progress has included identifying sex‐linked regions in several plants with homomorphic sex chromosomes, and some of these have been found to be small parts of the chromosome pairs, allowing sex determining genes to be identified (e.g., Akagi et al., 2019; Harkess et al., 2017, 2020; Müller et al., 2020; Zhou, Macaya‐Sanz, Carlson, et al., 2020); the genes are often involved in hormone response pathways, mainly associated with cytokinin and ethylene response pathways (reviewed by Feng et al., 2020). XX/XY (male heterogametic) and ZW/ZZ (female heterogametic) sex determination systems have been found in close relatives (Balounova et al., 2019; Martin et al., 2019; Müller et al., 2020; Zhou, Macaya‐Sanz, Carlson, et al., 2020). The extent to which related dioecious plants share the same sex‐determining systems, or evolved dioecy independently, is still not well understood, although there is accumulating evidence for independent evolution in the Salicaceae (Yang et al., 2020).

After recombination stops between an evolving sex chromosome pair, or part of the pair, forming a fully sex‐linked region, repetitive sequences and transposable elements are predicted to accumulate rapidly (reviewed in Bergero & Charlesworth, 2009). The expected accumulation has been detected in both Y‐ and W‐linked regions of several plants with heteromorphic sex chromosome pairs (reviewed by Hobza et al., 2015). Repeat accumulation is also expected in X‐ and Z‐linked regions; although this is expected to occur to a much smaller extent, it has been detected in *Carica papaya* and *Rumex acetosa* (Gschwend et al., 2012; Jesionek et al., 2020; Wang, Na, et al., 2012). The accumulation of repeats reduces gene densities, compared with autosomal or pseudoautosomal regions (PARs), and this has been observed in *Silene latifolia*, again affecting both sex chromosomes (Blavet et al., 2015).

The accumulation of repetitive sequences is a predicted consequence of recombination suppression reducing the efficacy of selection in Y‐ and W‐linked regions compared to those carried on X and Z chromosomes, which also predicts that deleterious mutations will accumulate, causing Y and W chromosome genetic degeneration (reviewed by Charlesworth et al., 1994, Ellegren, 2011 and Wang, Na, et al., 2012). The chromosome that recombines in the homogametic sex (the X or Z) remains undegenerated and maintains the ancestral gene content of its progenitor chromosome, and purifying selection can act to maintain gene functions (Wilson & Makova, 2009). However, genes on these chromosomes are also predicted to evolve differently from autosomal genes. Compared with purifying selection acting on autosomal genes, hemizygosity of genes in degenerated regions increases the effectiveness of selection against X‐ or Z‐linked deleterious mutations (unless they are not expressed in the heterogametic sex, see Vicoso & Charlesworth, 2006). Positive selection may also act on X/Z‐linked genes, and will be particularly effective in causing spread of X‐linked male‐beneficial mutations (or Z in female‐beneficial ones in ZW systems), because mutations are hemizygous in the heterogametic sex (Vicoso & Charlesworth, 2006). When comparing coding sequences between different species, X‐ and Z‐linked genes may therefore have either higher *K*a/*K*s (nonsynonymous substitution per nonsynonymous site/synonymous substitution per synonymous site) ratios than autosomal genes, or lower ratios if purifying selection against deleterious mutations is more important (Vicoso & Charlesworth, 2006). Furthermore, X/Z‐linked regions may, over time, gain genes with beneficial effects in one sex but deleterious effects in the other (sexually antagonistic effects, see Arunkumar et al., 2009; Meisel et al., 2012; Rice, 1984).

Here, we investigated a previously unstudied member of the Salicaceae. The family *sensu lato* (*s*.*l*.) includes more than 50 genera and 1000 species, usually dioecious or monoecious (rarely hermaphroditic) (Chase et al., 2002; Cronk et al., 2015). Roughly half of the species are in two closely related genera of woody trees and shrubs, *Populus* and *Salix*, whose species are almost all dioecious (Argus, 2010; Fang et al., 1999), which might suggest that dioecy is the ancestral state. However, studies over the past 6 years, summarized in Table 1, show that the sex‐linked regions are located in different genome regions in different species, and that both genera include species whose sex‐determining regions (SDRs) appear to be in the early stages in the evolution.

**TABLE 1:** Summary of current information about sex‐linked regions in *Populus* and *Salix*

Taxon	Species	Male or female heterogamety	Chromosome carrying the sex‐determining locus	Estimated size of the sex‐linked regions (kb)	References
*Populus* (poplars)	*P. balsamifera*	Male	19	~100 (Y)	Geraldes et al. (2015); McKown et al. (2017)
	*P. deltoides*	Male	19	~300 (X, Y)	Xue et al. (2020)
	*P. euphratica*	Male	14	~84 (X), 658 (Y)	Yang et al. (2020)
	*P. nigra*	Male	19	Unknown	Gaudet et al. (2008);
	*P. tremula*	Male	19	~1000 (Y)	Müller et al. (2020)
	*P. trichocarpa*	Male	19	~100 (Y)	Geraldes et al. (2015); McKown et al. (2017); Zhou, Macaya‐Sanz, Schmutz, et al. (2020))
	*P. tremuloides*	Male	19	2000 (Y)	Pakull et al. (2009); Kersten et al. (2014)
	*P. alba*	Female	19	~140 (W), 33 (Z)	Müller et al. (2020); Yang et al. (2020)
*Salix* (willows)	subgenus *Salix* clade				
	*S. dunnii*	Male	7	3205 (X)	This study
	*S. nigra*	Male	7	2000	Sanderson et al. (2021)
	section *Amygdalinae*				
	*S. triandra*	Female	15	~6500	Li et al. (2020)
	*Chamaetia*‐*Vetrix* clade				
	*S. purpurea*	Female	15	6800 (W), 4000 (Z)	Zhou, Macaya‐Sanz, Carlson, et al. (2020))
	*S. suchowensis*	Female	15	Unknown	Hou et al. (2015)
	*S. viminalis*	Female	15	3100–3400 (W, Z)	Almeida et al. (2020)

*Populus* species usually have XX/XY systems and SDRs on chromosome 14 or 19, though a few species have ZW/ZZ systems with the SDRs also on chromosome 19. Until recently, all willows investigated were from one *Salix* clade, *Chamaetia*‐*Vetrix* (Lauron‐Moreau et al., 2015; Wu et al., 2015), and all were found to have female heterogamety and SDRs on chromosome 15 (Table 1), as does the close relative *Salix triandra* (section *Amygdalinae*), but, as the table shows, a recent study suggested an XX/XY system on chromosome 7 in *S. nigra*, the only species so far studied from the subgenus *Salix* clade (*sensu* Wu et al., 2015). This evidence for changes in the location of the sex‐linked regions, and for differences in the heterozygous sex, make the family Salicaceae interesting for studying the evolution of sex chromosomes, and in particular sex chromosome turnover.

To understand the evolutionary events involved in these differences, high‐quality genome sequences are needed, leading, potentially, to discovery of the sex‐determining gene(s), which can reveal whether the same gene is involved in species with the same heterogamety (perhaps even across different genera), or whether different lineages have independently evolved sex‐determining systems. Recent studies in *Populus* identified a member of the *Arabidopsis thaliana* Type A response regulator family (resembling ARABIDOPSIS RESPONSE REGULATOR 17, and therefore named *ARR17*), within the sex‐linked region on chromosome 19 of both *Populus tremula* and *P. deltoides*. This gene has been shown to be involved in sex‐determination in *P. tremula* and *P. deltoides* (Müller et al., 2020; Xue et al., 2020). In two species of the *Salix Chamaetia*‐*Vetrix* clade (*S. purpurea* and *S. viminalis*), an *ARR17*‐like gene is again detected in the W‐linked region (which is on a different chromosome, 15), and a partial and nonfunctional copy was also found in the Z‐linked region of the *S. purpurea* chromosome 15 (Almeida et al., 2020; Yang et al., 2020; Zhou, Macaya‐Sanz, Carlson, et al., 2020). Studying other willow species might confirm the presence of such a gene in all willow SDRs, or might instead find that some species' SDRs include no such gene. Species with different heterogamety are of particular interest, because it seems unlikely that the same gene could be male‐determining in male heterogamety, and female‐determining in a species with female heterogamety.

Although *Salix* is the largest genus in the family Salicaceae *s*.*l*., with ~450 species (reviewed in He et al., 2021), fewer *Salix* than *Populus* genomes have been assembled, and assemblies include only the cushion shrub *S. brachista* and the shrub willows *S. purpurea*, *S. suchowensis* and *S. viminalis* (Almeida et al., 2020; Chen et al., 2019; Wei et al., 2020; Zhou, Macaya‐Sanz, Carlson, et al., 2020). Shrub stature is a derived character, and the tree habit is ancestral (Skvortsov, 1999), and is usual in poplars.

Here, we describe studies in *S. dunnii*, a riparian willow tree of the subgenus *Salix* clade (*sensu* Wu et al., 2015), found in subtropical areas of China that can grow up to 10 m (Fang et al., 1999). Our study has three aims. First, we aim to develop a high‐quality, chromosome‐level assembly of the *S. dunnii* genome, which has not previously been sequenced. Second, we resequence samples of both sexes from natural populations to test whether this subgenus *Salix* species has an XX/XY system, and, if so, whether it is on chromosome 7, as in *S. nigra*, suggesting a possible independent evolutionary origin from the ZW systems in other *Salix* clades. Third, we study the evolution of the X‐linked region. Several interesting questions include (i) whether recombination in the region has changed since it became an X‐linked region (vs. an SDR having evolved within an already nonrecombining region), (ii) whether the genes in the region are orthologues of those in the homologous region of related species (vs. genes having been gained by movements from other genome regions), (iii) whether genes of the X‐linked region differ in expression between the sexes, and/or (iv) have undergone adaptive changes more often than other genes.

## 2 MATERIALS AND METHODS

### 2.1 Plant material

We collected young leaves from a female *Salix dunnii* plant (FAFU‐HL‐1) for genome sequencing. Silica‐gel‐dried leaves were used to estimate ploidy. Young leaf, catkin, stem and root samples for transcriptome sequencing were collected from FAFU‐HL‐1, and catkins and leaves from two other female and three male plants. We sampled 38 individuals from two wild populations of *S. dunnii* for resequencing. The plant material was frozen in liquid nitrogen and stored at −80°C until total genomic DNA or RNA extraction. For sequencing involving Oxford Nanopore Technologies (ONT) and Hi‐C, fresh leaf material was used. Table S1 gives detailed information about all the samples.

### 2.2 Ploidy determination

The ploidy of FAFU‐HL‐1 was measured by flow cytometry (FCM), using a species of known ploidy (*Salix integra*; 2*x* = 2*n* = 38, Wagner et al., 2020) as an external standard. The assay followed the FCM protocol of Doležel et al. (2007) (see Note S1).

### 2.3 Genome sequencing

For Illumina PCR‐free sequencing, total genomic DNA of FAFU‐HL‐1 was extracted using a Qiagen DNeasy Plant Mini kit following the manufacturer's instructions (Qiagen). For ONT sequencing, phenol–chloroform was used to extract DNA. PCR‐free sequencing libraries were generated using the Illumina TruSeq DNA PCR‐Free Library Preparation Kit (Illumina) following the manufacturer's recommendations. After quality assessment on an Agilent Bioanalyzer 2100 system, the libraries were sequenced on an Illumina platform (NovaSeq 6000) by Beijing Novogene Bioinformatics Technology (hereafter Novogene). ONT libraries were prepared following the Oxford Nanopore 1D Genomic DNA (SQKLSK109)‐PromethION ligation protocol, and sequenced by Novogene.

### 2.4 Hi‐C library preparation and sequencing

The Hi‐C library was prepared following a standard procedure (Wang et al., 2020). In brief, fresh leaves from FAFU‐HL‐1 were fixed with a 1% formaldehyde solution in MS buffer. Subsequently, cross‐linked DNA was isolated from nuclei. The *Dpn*II restriction enzyme was then used to digest the DNA, and the digested fragments were labelled with biotin, purified and ligated before sequencing. Hi‐C libraries were controlled for quality and sequenced on an Illumina Hiseq X Ten platform by Novogene.

### 2.5 RNA extraction and library preparation

Total RNA was extracted from young leaves, female catkins, stems and roots of FAFU‐HL‐1 using the Plant RNA Purification Reagent (Invitrogen) according to the manufacturer's instructions. Genomic DNA was removed using DNase I (TaKara). An RNA‐seq transcriptome library was prepared using the TruSeq RNA sample preparation Kit from Illumina, and sequencing was performed on an Illumina Novaseq 6000 by the Shanghai Majorbio Bio‐pharm Biotechnology (hereafter Majorbio).

### 2.6 Genome size estimation

The genome size was estimated by 17‐*k*‐mer analysis based on PCR‐free Illumina short reads to be ~376 Mb. Briefly, *k*‐mers were counted using jellyfish (Marçais & Kingsford, 2011), and the numbers used to estimate the genome size and repeat content using findgse (Sun et al., 2018). The proportion of sites in this individual that are heterozygous was estimated using genomescope (Vurture et al., 2017).

### 2.7 Genome assembly

smartdenovo (https://github.com/ruanjue/smartdenovo) and wtdbg2 (Ruan & Li, 2020) were used to create a *de novo* assembly based on ONT reads, using the following options: ‐c l to generate a consensus sequence, ‐J 5000 to remove sequences <5 kb, and ‐k 20 to use 20‐mers. We then selected the assembly with the highest N50 value and a genome size close to the estimated one, which was assembled by smartdenovo with canu correction (Koren et al., 2017) (Table S2). Since ONT reads contain systematic errors in regions with homopolymers, we mapped Illumina short reads to the genome and polished using pilon (Walker et al., 2014). The Illumina short reads were filtered using fastp (Chen et al., 2018) to remove adapters and sequences with low base quality before mapping.

### 2.8 Scaffolding with Hi‐C data

We filtered Hi‐C reads using fastp (Chen et al., 2018), then mapped the clean reads to the assembled genome with juicer (Durand et al., 2016), and finally assembled them using the 3d‐DNA pipeline (Dudchenko et al., 2017). Using juicebox (Durand et al., 2016), we manually cut the boundaries of chromosomes. To decrease the influence of interchromosome interactions and improve the chromosome‐scale assembly, we separately rescaffolded each chromosome with 3d‐DNA, and further corrected misjoins, order and orientation of a candidate chromosome‐length assembly using juicebox. Finally, we anchored the contigs to 19 chromosomes. The *Rabl* configuration (Dong & Jiang, 1998; Prieto et al., 2004) is not clear enough for reliable prediction of the centromere position in chromosome 7 of *S. dunnii* (Figure S1). As an alternative, we employed minimap2 (Li, 2018) with parameters “‐x asm20,” to identify the region with highest repeat sequence densities in the genome, which may represent the centromere.

### 2.9 Optimizing the genome assembly

To further improve the genome assembly, lr_gapcloser (Xu, Xu, et al., 2019) was employed twice for gap closing with ONT reads. We also used nextpolish (Hu et al., 2020) to polish the assembly, with three iterations with Illumina short reads to improve base accuracy. We subsequently removed contigs with identity of more than 90% and overlap of more than 80%, which were regarded as redundant sequences, using redundans (Pryszcz & Gabaldón, 2016). Overall, we removed a total of 8.62 Mb (40 contigs) of redundant sequences. Redundant sequences were mainly from the same regions of homeologous chromosomes (Pryszcz & Gabaldón, 2016). To identify and remove contaminating sequences from other species, we used the contigs to blast against the NCBI‐NT database, and found no contaminated contigs.

### 2.10 Characterization of repetitive sequences

Repeat elements were identified and classified using repeatmodeler (http://www.repeatmasker.org/) to produce a repeat library. Then repeatmasker was used to identify repeated regions in the genome, based on the library. The repeat‐masked genome was subsequently used in gene annotation.

### 2.11 Annotation of full‐length LTR‐RTs and estimation of insertion times

We annotated full‐length long terminal repeat retrotransposons (LTR‐RTs) in our assembly and estimated their insertion times as described in Xu, Liu, et al. (2019). Briefly, ltrharvest (Ellinghaus et al., 2008) and ltrdigest (Steinbiss et al., 2009) were used to *de novo* predict full‐length LTR‐RTs in our assembly. LTR‐RTs were then extracted and compared with *Gag*‐*Pol* protein sequences within the REXdb database (Neumann et al., 2019). To estimate their insertion times, the LTRs of individual transposon insertions were aligned using mafft (Katoh & Standley, 2013), and divergence between the 5′ and 3′‐LTR was estimated (Ma & Bennetzen, 2004; SanMiguel et al., 1998). The divergence values were corrected for saturation by Kimura's 2‐parameter method (Kimura, 1980), and insertion times were estimated from the values, assuming a mutation rate of 2.5 × 10^−9^ substitutions year^−1^ per site (Ingvarsson, 2008).

### 2.12 Transcriptome assembly and gene annotation

The genome was annotated by combining evidence from transcriptome, *ab initio* prediction and protein homology based on prediction. pasa (Program to Assemble Spliced Alignment, Haas et al., 2003) was used to obtain high‐quality loci based on transcriptome data. We randomly selected half of these loci as a training data set to train the augustus (Stanke et al., 2008) gene modeller, and the other half as the test data set, and conducted five replicates of optimization. The high‐quality loci data set was also used to train snap (Korf, 2004). A total of 103,540 protein sequences were obtained from *Arabidopsis thaliana*, *P. trichocarpa*, *S. purpurea* and *S. suchowensis* and used as reference proteins for homology‐based gene annotation. Gene annotation was then performed with the maker pipeline (Cantarel et al., 2008) (detail process presented in Note S2).

To annotate tRNA and rRNA sequences, we used trnascan‐se (Lowe & Eddy, 1997) and rnammer (Lagesen et al., 2007), respectively, and other noncoding RNAs (ncRNAs) were identified by querying against the Rfam database (Nawrocki et al., 2015).

For protein functional annotation, the annotated genes were aligned to proteins in the Uniprot database (including the SWISS‐PROT and TrEMBL databases, https://www.uniprot.org/), NR (https://www.ncbi.nlm.nih.gov/), Pfam and eggNOG (Powell et al., 2014) databases using blat (*E* value <10^−5^) (Kent, 2002). Motifs and functional domains were identified by searching against various domain libraries (ProDom, PRINTS, Pfam, SMART, PANTHER and PROSITE) using interproscan (Jones et al., 2014). Annotations were also assigned to GO (http://geneontology.org/) and KEGG (https://www.genome.jp/kegg/pathway.html) metabolic pathways to obtain more functional information.

To identify pseudogenes, the proteins were aligned against the genome sequence using tblastn with parameter settings of “‐m 8 ‐e 1e‐5.” pseudopipe with default parameter settings was then used to detect pseudogenes in the whole genome (Zhang et al., 2006).

### 2.13 Comparative phylogenetic analysis across willows

We performed a comparative genomic investigation of the available willow genomes (*Salix dunnii*, *S. brachista*, *S. purpurea*, *S. suchowensis* and *S. viminalis*), using *Populus trichocarpa* as an outgroup (Table S3). orthofinder2 (Emms & Kelly, 2019) was used to identify groups of orthologous genes. A maximum likelihood (ML) phylogenetic tree was constructed using iq‐tree (Nguyen et al., 2015) based on single‐copy orthologues extracted from orthogroups. The CDS (coding DNA sequence) of the single‐copy orthologous genes identified were aligned with mafft (Katoh & Standley, 2013), and then trimmed with trimai (Capella‐Gutiérrez et al., 2009). Finally, mcmctree in paml (Yang, 2007) was used to estimate the divergence time. For more details, see Note S3. We performed collinearity analysis of *P. trichocarpa* and the five willows, and self‐comparison of each species, using mcscanx with the default parameters (Wang, Tang, et al., 2012). kaks_calculator (Wang et al., 2010) was used to calculate *K*s values, based on orthologous pairs, using the Yang–Nielsen (YN) model (Zhang & Yu, 2006).

### 2.14 Whole‐genome resequencing and SNP calling

Total genomic DNA for all 38 samples from natural populations (Table S1) was extracted with the Qiagen DNeasy Plant Mini Kit (Qiagen) following the manufacturer's instructions. Whole‐genome resequencing using paired‐end libraries was performed on an Illumina NovaSeq 6000 by Majorbio. The sequenced reads were filtered and trimmed by fastp (Chen et al., 2018). The filtered reads were then aligned to the assembled genome using the BWA‐MEM algorithm from bwa (Li, 2013; Li & Durbin, 2009). samtools (Li et al., 2009) was used to extract primary alignments, sort, and merge the mapped data. sambamba (Tarasov et al., 2015) was used to mark potential duplications in the PCR amplification step of library preparation. Finally, freebayes (Garrison & Marth, 2012) was employed for single‐nucleotide polymorphism (SNP) calling, yielding 10,985,651 SNPs. vcftools (Danecek et al., 2011) was used to select high‐quality SNPs based on the calling results: we (i) excluded all genotypes with a quality below 20, (ii) included only genotypes with coverage depth of at least 5 and not more than 200, (iii) retained only bi‐allelic SNPs, and (iv) removed SNPs with missing information rate >20% and minor allele frequency <5%. This yielded 4,370,362 high‐quality SNPs for analysis.

### 2.15 Identification of the sex determination system in *S. dunnii*

We used our high‐quality SNPs in a standard case‐control genome‐wide association study (GWAS) between allele frequencies and sex phenotype using plink (Purcell et al., 2007). SNPs with α < 0.05 after Bonferroni correction for multiple testing were considered significantly associated with sex.

The chromosome quotient (CQ) method (Hall et al., 2013) was employed to further test whether *S. dunnii* has a female or male heterogametic system. The CQ is the normalized ratio of female to male alignments to a given reference sequence, using the stringent criterion that the entire read must align with zero mismatches. To avoid bias due to different numbers of males and females, we used only 18 individuals of each sex (Table S1). We filtered the reads with fastp, and made combined female and male read data sets. The cq‐calculate.pl software (https://sourceforge.net/projects/cqcalculate/files/CQ‐calculate.pl/download) was used to calculate the CQ for each 50‐kb nonoverlapping window of the *S. dunnii* genome. For male heterogamety, we expect a CQ value close to 2 in windows in the X‐linked region (denoted below by X‐LR), given a female genome sequence, whereas, for female heterogamety we expect CQ ≈ 0.5 for Z‐linked windows, and close to zero for W‐linked windows.

Population genetic statistics, including nucleotide diversity per base pair (π) and observed heterozygote frequencies (*H*_O_) were calculated for female and male populations using vcftools (Danecek et al., 2011) or the “populations” module in stacks (Catchen et al., 2011). Weighted *F*_ST_ values between the sexes were calculated using the Weir and Cockerham (1984) estimator with 100‐kb windows and 5‐kb steps. A changepoint package (Killick & Eckley, 2014) was used to assess significance of differences in the mean and variance of the *F*_ST_ values between the sexes of chromosome 7 windows, using the function cpt.meanvar, algorithm PELT and penalty CROPS. poplddecay (Zhang et al., 2019) was used to estimate linkage disequilibrium (LD) based on unphased data, for the whole genome and the X‐LR, with parameters “‐MaxDist 300 ‐MAF 0.05 ‐Miss 0.2.” Furthermore, we retained 20 females from 38 individual data sets and obtained 60,848 SNPs separated by at least more than 5 kb, and employed ldblockshow (Dong et al., 2020) to calculate and visualize the LD pattern of each chromosome.

### 2.16 Gene content of chromosome 7 of *S. dunnii*

The Python version of mcscan (Tang et al., 2008) was used to analyse chromosome collinearity between the protein‐coding sequences detected in the whole genomes of *S. dunnii*, *S. purpurea* and *P. trichocarpa*. The “‐‐cscore=.99” was used to obtain reciprocal best hit (RBH) orthologues for synteny analysis.

To identify homologous gene pairs shared by chromosome 7 and the autosomes of *S. dunnii*, and those shared with chromosome 7 of *P. trichocarpa*, and *S. purpurea* (using the genome data in Table S3), we performed reciprocal blasts of all primary annotated peptide sequences with “blastp ‐evalue 1e‐5 ‐max_target_seqs 1.” For genes with multiple isoforms, only the longest one was used. Furthermore, homologues of *S. dunnii* chromosome 7 genes in *Arabidopsis thaliana* were identified with the same parameters.

Because the similar *A. thaliana ARR17* gene (Potri.019G133600; reviewed in Müller et al., 2020) has been proposed and confirmed to be involved in sex‐determination in *Populus* (see Introduction), we also blasted its sequence against our assembled genome with “tblastn ‐max_target_seqs 5 ‐evalue 1e‐5” to identify possible homologous intact or pseudogene copies.

### 2.17 Molecular evolution of chromosome 7 homologues of willow and poplar

To test whether X‐linked genes in our female genome sequence evolve differently from other genes, we aligned homologues of chromosome 7 sequences identified by blastp, and estimated the value of *K*a and *K*s between *S. dunnii* and *P. trichocarpa*, and between *S. dunnii* and *S. purpurea*. To obtain estimates for an autosome for the same species pairs, we repeated this analysis for chromosome 6 (this is the longest chromosome, apart from chromosome 16, which has a different arrangement in poplars and willows, see Results; Table S4). paraat (Zhang et al., 2012) and clustalw2 (Larkin et al., 2007) were used to align the sequences, and the yn00 package of paml (Yang, 2007) was used to calculate the *K*a and *K*s values for each homologous pair.

### 2.18 Gene expression

We used seqprep (https://github.com/jstjohn/SeqPrep) and sickle (https://github.com/najoshi/sickle) to trim and filter the raw data from 12 tissue samples (catkins and leaves from each of three female and male individuals) (Table S1).

Clean reads were separately mapped to our assembled genome for each sample using star (Dobin et al., 2013) with parameters “‐‐sjdbOverhang 150, ‐‐genomeSAindexNbases 13.” The featurecounts (Liao et al., 2014) was employed to merge different transcripts to a consensus transcriptome and calculate counts separately for each sex and tissue. Then we converted the read counts to TPM (transcripts per million reads), after filtering out unexpressed genes (counts=0 in all samples, excluding non‐mRNA). In total, 28,177 (89.45%) genes were used for subsequent analyses. The deseq2 package (Love et al., 2014) was used to detect genes differentially expressed in the different sample groups. The deseq default was used to test differential expression using negative binomial generalized linear models and estimation of dispersion and logarithmic fold changes incorporating data‐driven prior distributions, to yield log_2_FoldChange values and *p* values adjusted for multiple tests (adjusted *p* value <.05, |log_2_FoldChange| (absolute value of log_2_FoldChange) > 1).

## 3 RESULTS

### 3.1 Genome assembly

The *k*‐mer analysis of our sequenced genome of a female *Salix dunnii* plant indicated that the frequency of heterozygous sites in this diploid individual is low (0.79%) (Figures S2 and S3; Table S1). We generated 72 Gb (~180×) of ONT long reads, 60 Gb (~150×) Illumina reads and 55 Gb (~140×) of Hi‐C reads (Tables S5 and S6). After applying several different assembly strategies, we selected the one with the “best” contiguity metrics (smartdenovo with canu correction, Table S2). Polishing/correcting using Illumina short reads of the same individual yielded a 333‐Mb genome assembly in 100 contigs (contig N50 = 10.1 Mb) (Table S2).

**FIGURE 1:**
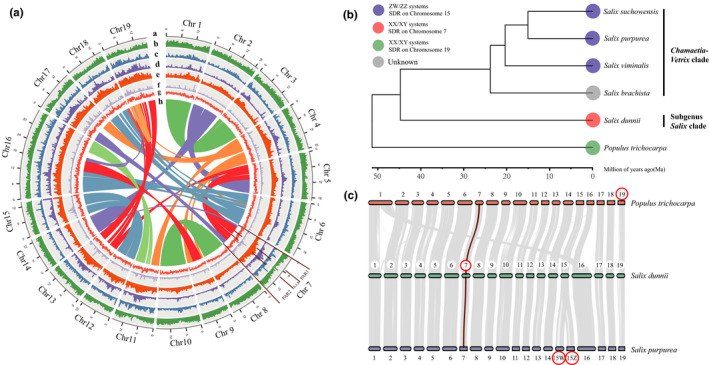
Genome structure and evolution of *Salix dunnii*. a, circos plot showing: (a) the chromosome lengths in Mb, (b) gene density, (c) LTR‐Copia density, (d) LTR‐Gypsy density, (e) total repeats, (f) density of pseudogenes, (g) GC (guanine‐cytosine) content and (h) syntenic blocks. b, Inferred phylogenetic tree of *S. brachista*, *S. dunnii*, *S. purpurea*, *S. suchowensis*, *S. viminalis* and the outgroup *Populus trichocarpa*, with divergence times. The root age of the tree was calibrated to 48–52 million years ago (Ma) following Chen et al. (2019) and the crown age of the *Chamaetia*‐*Vetrix* clade (here including *S. brachista*, *S. purpurea*, *S. suchowensis*, and *S. viminalis*) was calibrated to 23–25 Ma according to Wu et al. (2015). c, Macrosynteny between genomic regions of *P. trichocarpa*, *S. dunnii* and *S. purpurea*. The dark orange line shows the syntenic regions between the *S. dunnii* X‐linked region of chromosome 7, and the homologous regions in the same chromosomes of *S. purpurea* and *P. trichocarpa*. Red circles show the chromosomes carrying sex‐linked regions

With the help of Hi‐C scaffolding, we achieved a final chromosome‐scale assembly of 328 Mb of 29 scaffolds (scaffold N50 = 17.28 Mb), about 325.35 Mb (99.17%) of which is anchored to 19 pseudochromosomes (Figure 1a, Table 2; Figure S4, Table S4), corresponding to the haploid chromosome number of the species. The mitochondrial and chloroplast genomes were assembled into circular DNA molecules of 711,422 and 155,620 bp, respectively (Figures S5 and S6). About 98.4% of our Illumina short reads were successfully mapped back to the genome assembly, and about 99.5% of the assembly was covered by at least 20× reads. Similarly, 98.9% of ONT reads mapped back to the genome assembly and 99.9% were covered by at least 20× reads. The assembly's LTR Assembly Index (LAI) score was 12.7, indicating that our assembly reached a high enough quality to achieve the rank of “reference” (Ou et al., 2018). busco (Simão et al., 2015) analysis identified 1392 (96.6%) of the 1440 highly conserved core proteins in the Embryophyta database, of which 1239 (86.0%) were single‐copy genes and 153 (10.6%) were duplicate genes. A further 33 (2.3%) had fragmented matches to other conserved genes, and 37 (2.6%) were missing.

**TABLE 2:** Statistics of the *Salix dunnii* genome assembly

Total assembly size (Mb)	328
Total number of contigs	31
Total anchored size (Mb)	325.352
Maximum contig length (Mb)	35.892
Minimum contig length (kb)	68.49
Contig N50 length (Mb)	16.657
Contig L50 count	8
Contig N90 length (Mb)	12.795
Contig L90 count	17
Total number of scaffolds	29
Maximum scaffold length (Mb)	35.892
Minimum scaffold length (kb)	68.49
Scaffold N50 length (Mb)	17.281
Scaffold L50 count	8
Scaffold N90 length (Mb)	13.179
Scaffold L90 count	17
Gap number	2
GC content (%)	33.09
Gene number	31,501
Repeat content (%)	41.05

### 3.2 Annotation of genes and repeats

In total, 134.68 Mb (41.0%) of the assembled genome consisted of repetitive regions (Table 2), close to the 41.4% predicted by findgse (Sun et al., 2018). LTR‐RTs were the most abundant annotations, forming up to 19.1% of the genome, with *Gypsy* and *Copia* class I retrotransposon (RT) transposable elements (TEs) accounting for 13% and 5.85% of the genome, respectively (Table S7). All genomes so far studied in *Salix* species have considerable proportions of TE sequences, but the higher proportions of *Gypsy* elements in *S. dunnii* (Table S7) (Chen et al., 2019) suggested considerable expansion in this species. Based on estimated divergence per site (see Methods), most full‐length LTR‐RTs appear to have inserted at different times within the last 30 million years rather than in a recent burst (Figures S7‐S9; Table S8). Divergence values of all chromosomes are 0 to 0.2, mean 0.041 and median 0.027. The values for just chromosome 7 are similar, range from 0 to 0.18, but the mean 0.0461 and median 0.035 slightly higher than for the chromosomes other than 7, and this is mainly caused by a higher value/greater age in the X‐linked region.

Using a comprehensive strategy combining evidence‐based and *ab initio* gene prediction (see Methods), we then annotated the repeat‐masked genome. We identified a total of 31,501 gene models, including 30,200 protein‐coding genes, 650 transfer RNAs (tRNAs), 156 ribosomal RNAs (rRNA) and 495 unclassifiable noncoding RNAs (ncRNAs) (Table 2; Table S9). The average *S. dunnii* gene is 4095.84 bp long and contains 6.07 exons (Table S10). Most of the predicted protein‐coding genes (94.68%) matched a predicted protein in a public database (Table S11). Among the protein‐coding genes, 2053 transcription factor (TF) genes were predicted and classified into 58 gene families (Tables S12 and S13).

### 3.3 Comparative genomics and whole genome duplication events

We compared the *S. dunnii* genome sequence to four published willow genomes and *Populus trichocarpa*, as an outgroup, using 5950 single‐copy genes to construct a phylogenetic tree of the species' relationships (Figure 1b). Consistent with published topologies (Wu et al., 2015), *S. dunnii* appears in our study as an early diverging taxon in sister position to the four *Salix* species of the *Chamaetia*‐*Vetrix* clade.

To test for whole genome duplication (WGD) events, we examined the distribution of *K*s values between paralogues within the *S. dunnii* genome, together with a dot plot to detect potentially syntenic regions. This revealed a *K*s peak similar to that observed in *Populus*, confirming the previous conclusion that a WGD occurred before the two genera diverged (*K*s around 0.3 in Figure S10) (Tuskan et al., 2006). A WGD is also supported by our synteny analysis within *S. dunnii* (Figure 1a; Figure S11). Synteny and collinearity were nevertheless high between *S. dunnii* and *S. purpurea* on all 19 chromosomes, and between the two willow species and *P. trichocarpa* for 17 chromosomes (Figure 1c), with a previously known large interchromosomal rearrangement between chromosome 1 and chromosome 16 of *Salix* and *Populus* (Figure 1c).

### 3.4 Identification of the sex determination system

To infer the sex determination system in *S. dunnii*, we sequenced 20 females and 18 males from two wild populations by Illumina short‐read sequencing (Table S1). After filtering, we obtained more than 10 Gb of clean reads per sample (Table S14) with average depths of 30× to 40× (Table S15), yielding 4,370,362 high‐quality SNPs.

**FIGURE 2:**
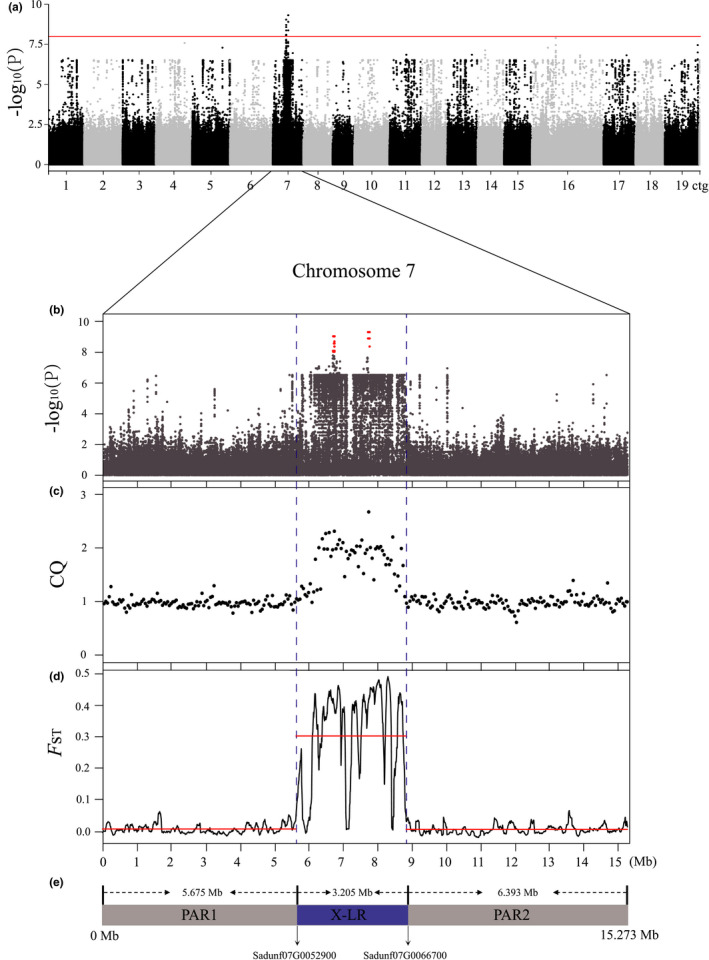
Identification of the sex‐determination systems of *Salix dunnii*. (a) Results of genome wide association studies (GWAS) between SNPs and sexes in 38 individuals. The *y* axis is the negative logarithm of *p* values, and the red line shows the Bonferroni‐corrected significance level corresponding to α < 0.05. (b) Manhattan plot for GWAS *p*‐values of all SNPs of chromosome 7. Red dots show significantly sex‐associated SNPs. (c) Chromosome quotients (CQ) in 50‐kb nonoverlapping window of chromosome 7. (d) *F*_ST_ values between the sexes for 100‐kb overlapping windows of chromosome 7 calculated at 5‐kb steps. Red lines represent three significant regions on chromosome 7 suggested by changepoint analysis. (e) The positions of PAR1, X‐LR and PAR2 of chromosome 7

A GWAS revealed a small (1,067,232 bp) *S. dunnii* chromosome 7 region, between 6,686,577 and 7,753,809 bp, in which 101 SNPs were significantly associated with sex (Figure 2a,b; Table S16, Figure S12). More than 99% of these candidate sex‐linked SNPs are homozygous in all the females, and 63.74% are heterozygous in all the males in our sample (Table S17).

Consistent with our GWAS, the CQ method, with 18 individuals of each sex, detected the same region, and estimated a somewhat larger region, between 6.2 and 8.75 Mb, with CQ > 1.6 (which includes all the candidate sex‐linked SNPs), whereas other regions of chromosome 7 and the other 18 chromosomes and contigs have CQ values close to 1 (Figure 2c; Figure S13). These results suggest that *S. dunnii* has a male heterogametic system, with a small completely sex‐linked region on chromosome 7. Because these positions are based on sequencing a female, and the species has male heterogamety, we refer to this as the X‐linked region (X‐LR). We predicted (see Methods) that the chromosome 7 centromere lies between roughly 5.2 and 7.9 Mb, implying that the sex‐linked region may be in a low recombination region near this centromere (Figure S1). Moreover, the analysis of LD using 20 females shows that the X‐LR is located within a region of the X chromosome with lower recombination than the rest of chromosome 7, consistent with a centromeric or pericentromeric location (Figure S14). Without genetic maps, it is not yet clear whether this species has low recombination near the centromeres of all its chromosomes.

Genetic differentiation (estimated as *F*_ST_) between our samples of male and female individuals further confirmed a 3.205‐Mb X‐LR region in the region detected by the GWAS. Between 5.675 and 8.88 Mb (21% of chromosome 7), changepoint analysis (see Methods) detected *F*_ST_ values significantly higher than those in the flanking regions, as expected for a completely X‐linked region (Figure 2; Figure S15). The other 79% of the chromosome forms two PARs (see Figure 2). LD was substantially greater in the putatively fully sex‐linked region than in the whole genome (Figure S16).

### 3.5 Gene content of the fully sex‐linked region

We found 124 apparently functional genes in the X‐LR (based on intact coding sequences) vs. 516 in PAR1 (defined as the chromosome 7 region from position 0 to 5,674,999 bp), and 562 in PAR2 in chromosome 7 (from 8,880,001 to 15,272,728 bp) (Figure 2e; Tables S9 and S18). The X‐LR gene numbers are only 10.3% of the functional genes on chromosome 7, vs. 21% of its physical size, suggesting either a low gene density or loss of function of genes, either of which could occur in a pericentromeric genome region. We also identified 183 X‐linked pseudogenes. Including pseudogenes, X‐LR genes form 17% of this chromosome's gene content, and therefore overall gene density is not much lower than in the PARs. Instead, pseudogenes form a much higher proportion (59%) than in the autosomes (31%), or the PARs (148 and 269 in PAR1 and in PAR2, respectively, or 28% overall, see Tables S19 and S20). In total, 41 genes within the X‐linked region had no blast hits on chromosome 7 of either *P. trichocarpa* or *S. purpurea* (Table S18).

Our searches of the *S. dunnii* genome for complete or partial copies of the Potri.019G133600 sequence (the *ARR17*‐like gene described above, and discussed further below, that is involved in sex‐determination on several other Salicaceae) found copies on chromosomes 1, 3, 8, 13 and 19 (Table S21). Importantly, we found none on chromosome 7, and specifically no copy or pseudogene copy in the X‐LR.

### 3.6 Molecular evolution of *S. dunnii* X‐linked genes

**FIGURE 3:**
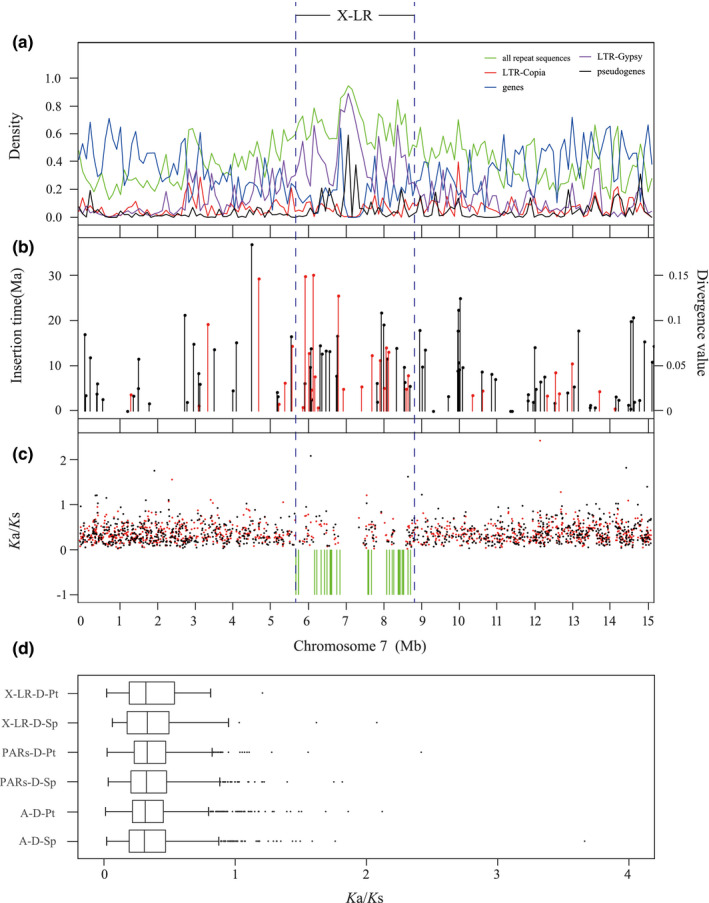
Analysis of *Salix dunnii* chromosome 7 genes. (a) Densities of two transposable element types, LTR‐Gypsy (purple line) and LTR‐Copia (red line), all repeat sequences (green line), pseudogenes (black line), as well as genes (blue line) in the entire chromosome 7 of *S. dunnii*. (b) Estimated insertion times and divergence values of full‐length long terminal repeat retrotransposons (LTR‐RTs) in chromosome 7 of *S. dunnii*. The red lines represent LTR‐Gypsy, and the black lines LTR‐Copia elements. (c) Comparison of *K*a/*K*s ratios between homologous genes in *S. dunnii* and *Populus trichocarpa* (red dots), and of *S. dunnii* vs. *S. purpurea* (black dots). Green lines indicate locations of *S. dunnii* X‐linked genes with no hits in either *S. purpurea* or *P. trichocarpa*. (d) Comparison of *K*a/*K*s values of X‐LR, PARs and autosomal genes (chromosome 6). X‐LR‐D‐Pt and PARs‐D‐Pt are obtained from the homologous genes of *S. dunnii* and *P. trichocarpa*. X‐LR‐D‐Sp and PARs‐D‐Sp are obtained from chromosome 7 of the homologous genes of chromosome 7 of *S. dunnii* and *S. purpurea*. A‐D‐Pt and A‐D‐Sp are obtained from the homologous genes of chromosome 6 of *S. dunnii* – *P. trichocarpa* (1897 homologous pairs) and *S. dunnii* – *S. purpurea* (1852 homologous pairs), respectively. The Wilcoxon rank sum test was used to detect significance differences of different regions of the two data sets. No significant difference (*p* < .05) was detected between the sex‐linked region and the autosomes or PARs (Figure S19)

Gene density is lower in the X‐LR than the PARs, probably because LTR‐Gypsy element density is higher (Figure 3a). Repetitive elements make up 70.58% of the X‐LR, vs. 40.36% for the PARs and 40.78% for the 18 autosomes (Table 3). More than half (53.31%) of the identified intact LTR‐Gypsy element of chromosome 7 were from X‐LR (Figure 3b; Table S8).

We estimated *K*a, *K*s and *K*a/*K*s ratios for chromosome 7 genes that are present in both *S. dunnii* and *S. purpurea* (992 orthologue pairs) or *S. dunnii* and *P. trichocarpa* (1017 orthologue pairs). Both *K*a and *K*s values are roughly similar across the whole chromosome (Figures S17 and S18), and the *K*a/*K*s values did not differ significantly between the sex‐linked region and the autosomes or PARs (Figure 3c,d; Figure S19). However, the *K*a and *K*s estimates for PAR genes are both significantly higher than for autosomal genes, suggesting a higher mutation rate (Figure S17 shows the results for divergence from *P. trichocarpa*, and Figure S18 for *S. purpurea*).

**TABLE 3:** Total size (Mb) of regions represented by genes and repeat sequences in different regions of the genome (all autosomes were compared with the chromosome 7 X‐linked region and its PARs); in parentheses are the proportions of the total lengths of the regions represented by each sequence type

Category	X‐LR	PARs	Autosomes
Genes	0.537 (16.77%)	4.679 (38.78%)	122.740 (39.58%)
Gypsy‐LTR	1.429 (44.60%)	1.370 (11.36%)	39.321 (12.68%)
Copia‐LTR	0.190 (5.94%)	0.844 (6.99%)	17.986 (5.80%)
Total repeats	2.262 (70.58%)	4.870 (40.36%)	126.465 (40.78%)

### 3.7 Sex‐biased gene expression in reproductive and vegetative tissues

**FIGURE 4:**
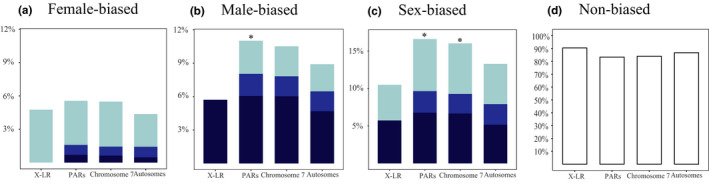
Distribution of sex‐biased (|log_2_FoldChange| > 1, adjusted *p* value <.05) and nonbiased expression genes in catkins. (a) Female‐biased genes. (b) Male‐biased genes. (c) Sex‐biased genes. (d) Nonbiased genes. The percentages of female‐biased, male‐biased or nonbiased expression genes are shown for different fold change categories (|log_2_FoldChange|). Light blue bars show values >1, blue indicate values >2, dark blue indicates >3, and open bars are changes less than or equal to two‐fold. Pearson's Chi‐squared test was used to test for significance differences of sex‐based expression genes in different regions (*p < .05)

After quality control and trimming, more than 80% of our RNAseq reads mapped uniquely to the genome assembly across all samples (Table S22). In both the catkin and leaf data sets, there are significantly more male‐ than female‐biased genes. In catkins, 3734 genes have sex differences in expression (2503 male‐ and 1231 female‐biased genes). Only 43 differentially expressed genes were detected in leaf material (31 male‐ vs. 12 female‐biased genes, mostly also differentially expressed in catkins; Figure S20, Table S23). Chromosome 7, as a whole, showed a similar enrichment for genes with male‐biased expression (117 male‐biased genes, out of 1112 that yielded expression estimates, or 10.52%), but male‐biased genes form significantly higher proportions only in the PARs, and not in the X‐linked region (Figure 4), which included only six male‐ and five female‐biased genes, while the other 94 X‐LR genes that yielded expression estimates (90%) were unbiased.

We divided genes into three groups according to their sex differences in expression, based on the log_2_FoldChange values. All the male biased X‐LR genes are in the higher expression category, but higher expression female‐biased genes are all from the PARs (Figure 4).

## 4 DISCUSSION

### 4.1 Chromosome‐scale genome assembly of *S. dunnii*

The assembled genome size of *Salix dunnii* is about 328 Mb (Table 2), similar to other willow genomes (which range from 303.8 to 357 Mb, Table S24). The base chromosome number for the Salicaceae *s*.*l*. family is *n* = 9 or 11, whereas the Salicaceae *sensu stricto* have a primary chromosome number of *n* = 19 (reviewed in Cronk et al., 2015). *Populus* and *Salix* underwent a palaeotetraploidy event that caused a change from *n* = 11 to *n* = 22 before the split from closely related genera of this family (e.g., *Idesia*), followed by reduction to *n* = 19 in *Populus* and *Salix* (Darlington & Wylie, 1955; Li et al., 2019; Xi et al., 2012). We confirmed that *Populus* and *Salix* share the same WGD (Figure S10a), and generally show high synteny and collinearity (Figure 1c).

### 4.2 A male heterogametic sex determination system in *S. dunnii*

The *S. dunnii* sex determination region is located on chromosome 7 (Figure 2), the same chromosome as the only other species previously studied in subgenus *Salix*, *S. nigra* (Sanderson et al., 2021). The size of the X‐linked region, 3.205 Mb, is similar to the sizes of Z‐linked regions of other willows (Table 1), and they are all longer than any known *Populus* X‐linked regions. These data support the view (Yang et al., 2020) that sex‐determining loci have probably evolved independently within the genus *Salix*, as well as separately in poplars. This is consistent with evidence that, despite dioecy being found in almost all willows, the W‐linked sequences of some species began diverging within the genus (Pucholt et al., 2017; Zhou, Macaya‐Sanz, Carlson, et al., 2020). A high‐quality assembly of the Y‐linked region of *S. dunnii* is planned, and should further aid our understanding of the evolution of sex determination systems in *Salix*.

### 4.3 Gene content evolution in the *S. dunnii* X‐linked region

Our synteny analyses and homologous gene identification for the X‐LR of our sequenced female support the independent evolution hypothesis (Figure 1c). Many *S. dunnii* X‐LR protein‐coding genes have homologues on chromosome 7 of *Populus trichocarpa* and/or *S. purpurea* (Table S18), showing that the region evolved from an ancestral chromosome 7 and was not translocated from another chromosome. However, a third of the protein‐coding genes were not found in even the closer outgroup species, *S. purpurea*, whose chromosome 7 is an autosome. These genes appear to have been duplicated into the region from other *S. dunnii* chromosomes, as follows: chromosome 16 (eight genes), 13 (six genes), 12 (four genes), 17 (four genes), 19 (four genes), and nine genes from other chromosomes (Table S18). Two of these genes (Sadunf07G0053500 and Sadunf07G0053600) are involved in reproductive processes (these reciprocal best hits found the *Arabidopsis thaliana* genes EMBRYO DEFECTIVE 3003, involved in embryo development and seed dormancy, and CLP‐SIMILAR PROTEIN 3, which is involved in flower development). Two other genes (Sadunf07G0059600 and Sadunf07G0059800) have sex‐biased expression (Table S18). However, we cannot conclude that these duplications were selectively advantageous, moving genes with reproductive functions to the X‐linked region, as an alternative cannot be excluded (see below).

Given the numerous genes in the *S. dunnii* X‐linked region, and the current lack of an assembled male genome sequence, no candidate sex‐determining gene can yet be proposed for this species. In several *Populus* species with male heterogamety, the sex‐determining gene is an *ARR17*‐like gene (Müller et al., 2020; Xue et al., 2020). Such a gene has been suggested to be the sex‐determining gene of all Salicaceae (Yang et al., 2020), based on the finding of a similar gene in the W‐linked regions of *S. viminalis* and *S. purpurea* (Almeida et al., 2020; Zhou, Macaya‐Sanz, Carlson, et al., 2020). No such gene is present in the Z‐linked region of *S. viminalis*, consistent with the finding in *Populus* species that the sex‐determining gene is carried only in the Y‐ and not the X‐linked region. Our results are consistent with this, as we found no copy or partial duplicate of such a gene in the *S. dunnii* X‐linked region. However, several similar sequences were found elsewhere in the *S. dunnii* genome. Given the current lack of information about the Y‐linked region in this species, we cannot exclude the possibility that a Y‐linked similar gene may exist in this species.

In diploid organisms, only the Y chromosomes are predicted to degenerate, because X chromosomes recombine in the XX females (reviewed in Charlesworth, 2015). However, X‐ as well as Y‐linked regions are expected to accumulate repetitive sequences to a greater extent than nonsex‐linked genome regions, due to their somewhat lower effective population size, and this has been detected in papaya and common sorrel (Jesionek et al., 2020; Wang, Na, et al., 2012). The *S. dunnii* X‐LR appears to have done the same, being rich in LTR‐Gypsy elements (Table 3; Figures 1a and 3a). As in papaya, it is not yet clear whether elements are enriched due to the region having become sex‐linked, or because of its location in the chromosome 7 pericentromeric region (Figure S1). The same uncertainty applies to the unexpectedly large numbers of pseudogenes (Table S20) and duplicated genes (Table S18) found in the X‐LR compared with other regions of the *S. dunnii* genome. However, insertions of these elements appear to have occurred after the genera *Populus* and *Salix* diverged (Figures 1b and 3b), about 48–52 million years ago (Chen et al., 2019). This suggests that either the centromere is not in the same position in both genera, or that accumulation has occurred since the region became sex‐linked.

It was unexpected to find that one‐third of the genes of *S. dunnii* X‐linked genes did not have orthologues on chromosome 7 of either *S. purpurea* or *P. trichocarpa* (Figure 3c; Table S18). These genes appear to have originated by duplications of genes on other *S. dunnii* chromosomes, and some of them may be functional in reproductive or sex‐specific processes. However, we did not detect generally elevated *K*a/*K*s ratios in the X‐linked region (Figure 3c,d; Figure S19), which would be expected for pseudogenes and nonfunctional gene duplicates, as well as for genes under adaptive changes that might be expected to occur in such a region. Possibly X‐linkage evolved too recently to detect such changes, or for many adaptive changes to have occurred, and therefore the picture indicates predominantly purifying selection, similar to the rest of the genome. Overall, the results suggest that TE accumulation may be an earlier change than other evolutionary changes, which is consistent with theoretical predictions that TEs can accumulate very fast (Maside et al., 2005). However, it is again unclear whether these changes are due to sex linkage, or to the region being pericentromeric.

### 4.4 Sex‐biased gene expression in reproductive and vegetative tissues

Sex‐biased gene expression may evolve in response to conflicting sex‐specific selection pressures (Connallon & Knowles, 2005). Our expression analysis revealed significantly more genes with male than female biases, mainly confirmed to genes expressed in catkins, and much less in leaf samples (Table S23). This is consistent with observations in other plant species (Muyle, 2019). Male‐biased genes were enriched in the *S. dunnii* PARs (Figure 4), but not in the fully X‐linked region (Figure 4), unlike the findings in *S. viminalis* (Pucholt et al., 2017) where male‐biased genes appeared to be mildly enriched in the sex‐linked region.

## AUTHOR CONTRIBUTIONS

Li He and Jian‐Feng Mao planned and designed the research. Li He, Kai‐Hua Jia, Ren‐Gang Zhang, Yuan Wang, Tian‐Le Shi, Zhi‐Chao Li, Si‐Wen Zeng, Xin‐Jie Cai, Aline Muyle, Ke Yang and Deborah Charlesworth analysed the data. Li He, Deborah Charlesworth, Kai‐Hua Jia, Yuan Wang, Ren‐Gang Zhang, Jian‐Feng Mao, Natascha Dorothea Wagner, Elvira Hörandl and Aline Muyle wrote the paper.

## Supporting information

NoteS1‐3&FigS1‐20

Table S1‐24

## Data Availability

This Whole Genome Shotgun project has been deposited at DDBJ/ENA/GenBank under the accession JADGMS000000000 (https://www.ncbi.nlm.nih.gov/nuccore/JADGMS000000000.1). The version described in this paper is version JADGMS010000000. Sequence data presented in this article can be downloaded from the NCBI database under BioProject accession PRJNA670558 (https://www.ncbi.nlm.nih.gov/bioproject/PRJNA670558).
