# Supplementary material for: Chromosome‐scale assembly of the genome of Salix dunnii reveals a male‐heterogametic sex determination system on chromosome 7
Source: Mol Ecol Resour. 2021 Mar 16;21(6):1966–82. doi: 10.1111/1755-0998.13362 (PMC8359994; doi:10.1111/1755-0998.13362)
Supplement: Supplementary file 1 — NoteS1‐3&FigS1‐20 [file MEN-21-1966-s002.docx]

**Supplementary Information for:**

**Chromosome-scale assembly of the genome of *Salix dunnii* reveals a male-heterogametic sex determination system on chromosome 7**

**Running title:** Male-heterogametic system in willow tree

Li He^1,2,✝,^*, Kai-Hua Jia^1,✝^, Ren-Gang Zhang^3^, Yuan Wang^2^, Tian-Le Shi^1^, Zhi-Chao Li^1^, Si-Wen Zeng^2^, Xin-Jie Cai^2^, Natascha Dorothea Wagner^4^, Elvira Hörandl^4^, Aline Muyle^5^, Ke Yang^6^, Deborah Charlesworth^6^, Jian-Feng Mao^1^*

**1** Beijing Advanced Innovation Center for Tree Breeding by Molecular Design, National Engineering Laboratory for Tree Breeding, Key Laboratory of Genetics and Breeding in Forest Trees and Ornamental Plants, Ministry of Education, College of Biological Sciences and Technology, Beijing Forestry University, Beijing, 100083, China

**2** College of Forestry, Fujian Agriculture and Forestry University, Fuzhou 350002, China

**3** Ori (Shandong) Gene Science and Technology Co., Ltd, Weifang, 261000, Shandong, China

**4** Department of Systematics, Biodiversity and Evolution of Plants (with Herbarium), University of Goettingen, 37073, Göttingen, Germany

**5** Department of Ecology and Evolutionary Biology, University of California Irvine, Irvine, 92697, CA, USA

**6** Institute of Evolutionary Biology, School of Biological Sciences, University of Edinburgh, West Mains Road, Edinburgh, EH93LF, UK

^✝^These authors contributed equally to this paper.

*Author for correspondence. Li He, e‐mail: [heli198724@163.com](mailto:heli198724@163.com); Jian-Feng Mao, e-mail: [jianfeng.mao@bjfu.edu.cn](mailto:jianfeng.mao@bjfu.edu.cn)

**Table of Contents:**

[Supplementary Note 3](#_Toc63253118)

[Note 1: Ploidy determination 3](#_Toc63253119)

[Note 2: Transcriptome assembly and gene annotation 3](#_Toc63253120)

[Note 3: Comparative phylogenetic analysis across willows 5](#_Toc63253121)

[Supplementary Figures 7](#_Toc63253122)

[Figure S1. 7](#_Toc63253123)

[Figure S2. 8](#_Toc63253124)

[Figure S3. 9](#_Toc63253125)

[Figure S4. 10](#_Toc63253126)

[Figure S5. 11](#_Toc63253127)

[Figure S6. 12](#_Toc63253128)

[Figure S7 13](#_Toc63253129)

[Figure S8*.* 14](#_Toc63253130)

[Figure S9*.* 15](#_Toc63253131)

[Figure S10. 16](#_Toc63253132)

[Figure S11. 17](#_Toc63253133)

[Figure S12. 18](#_Toc63253134)

[Figure S13. 19](#_Toc63253135)

[Figure S14. 20](#_Toc63253136)

[Figure S15. 21](#_Toc63253137)

[Figure S16. 22](#_Toc63253138)

[Figure S17. 23](#_Toc63253139)

[Figure S18. 24](#_Toc63253140)

[Figure S19. 25](#_Toc63253141)

[Figure S20. 26](#_Toc63253142)

[Supplementary Tables (Independent xlsx file named as Table S1-S24) 27](#_Toc63253143)

[Softwares 29](#_Toc63253168)

[Reference 32](#_Toc63253169)

# Supplementary Note

## Note 1: Ploidy determination

The ploidy of FAFU-HL-1 was measured by flow cytometry (FCM), using a species of known ploidy (*Salix integra*; 2x = 2n = 38, Wagner *et al.* 2020) as an external standard. The assay followed the FCM protocol of Doležel *et al.* (2007). About 20–50 mg of silica-gel dried leaf tissue was incubated for 80 min in 1 ml LB01 buffer, and then chopped with a razor blade. The cell culture was collected by gentle pipetting and filtered through a 38 µm nylon mesh. Before measurements, the samples were stained with 80μg ml^−1^ PI simultaneously with 80μg ml^−1^ RNase in an ice bath for 30 min. A total of about 5000 nuclei were measured for each sample using a MoFlo-XDP flow cytometer (Beckman Coulter, Inc., Indianapolis, United States). Ploidy level estimates were done as described in He *et al.* (2021).

## Note 2: Transcriptome assembly and gene annotation

RNA-seq reads were preprocessed with fastp (Chen *et al.* 2018) to remove adapters and low base quality sequences. The filtered RNA-seq reads were mapped to the assembled genome by HiSat2 (Kim *et al.* 2015), and then StringTie (Pertea *et al.* 2015) was used for reference-guided assembly and Trinity (Haas *et al.* 2013) for genome-guided assembly. Trinity was also used to directly *de novo* assemble the transcriptome based on the filtered reads. All assembled transcripts were combined, and sequences with >= 95% identity and >= 95% coverage were classified as redundant and removed using CD-HIT (Fu *et al.* 2012). This yielded a final transcriptome with a total of 142,615 sequences, which was used during subsequent gene annotation.

The genome was annotated by combining evidence from transcriptome, *ab initio* prediction, and protein homology based on prediction. PASA (Program to Assemble Spliced Alignment, Haas *et al.* 2003) was used to map transcriptome data to the repeat-masked genome, yielding a total of 24,007 high-quality loci. We randomly selected half of these loci as a training dataset to train the AUGUSTUS (Stanke *et al.* 2008) gene modeller, and the other half as the test dataset, and conducted five replicates of optimization. The high-quality loci data set was also used to train SNAP (Korf, 2004). The protein sequences of *Arabidopsis thaliana*, *P. trichocarpa*, *S. purpurea* and *S. suchowensis* were clustered, and processed to discard sequences with >= 95% identity and >= 95% coverage, using CD-HIT (sources of genome datasets used are listed in Table S3). A total of 103,540 protein sequences were obtained and used as reference proteins for homology-based gene annotation. Gene annotation was then performed with the MAKER pipeline (Cantarel *et al.* 2008). Finally, gene annotations with <= 50 amino acids, or with internal stop codons or uncertain bases, or no start or stop codon, were removed.

To annotate tRNA and rRNA sequences, we used tRNAScan-SE (Lowe & Eddy 1997) and RNAMMER (Lagesen *et al.* 2007), respectively, and other ncRNAs were identified by querying against the Rfam database (Nawrocki *et al.* 2015).

For protein functional annotation, the annotated genes were aligned to proteins in Uniprot database (including the SWISS-PROT and TrEMBL databases, https://www.uniprot.org/), NR (https://www.ncbi.nlm.nih.gov/), Pfam and eggNOG (Powell *et al.* 2014) databases using BLAT (E value <10^−5^) (Kent 2002). Motifs and functional domains were identified by searching against various domain libraries (ProDom, PRINTS, Pfam, SMART, PANTHER and PROSITE) using InterProScan (Jones *et al.* 2014). Annotations were also assigned to GO (http://geneontology.org/) and KEGG (https://www.genome.jp/kegg/pathway.html) metabolic pathways to obtain more functional information.

To identify pseudogenes, the proteins were aligned against the genome sequence using tBLASTn with parameter settings of “-m 8 -e 1e-5”. PseudoPipe with default parameter settings was then used to detect pseudogenes in the whole genome (Zhang *et al.* 2006).

## Note 3: Comparative phylogenetic analysis across willows

We performed a comparative genomic investigation of the available five willow genomes (*Salix dunnii*, *S. brachista*, *S. purpurea, S. suchowensis*, and *S. viminalis*), used *Populus trichocarpa* as an outgroup (Table S3). OrthoFinder2 (Emms & Kelly 2018) was used to identify groups of orthologous genes, based on the protein-coding sequences of the six species, and yielded a total of 35,856 orthogroups. We extracted 5,950 single-copy orthologs to construct a maximum likelihood (ML) phylogenetic tree with IQ-TREE (Nguyen *et al.* 2014) with the best-fit model (JTT+F+R6). The CDS (Coding DNA Sequence) of the single-copy orthologous genes identified were aligned with MAFFT (Katoh & Standley 2013), and then trimmed with tirmAI (Capella-Gutiérrez *et al.* 2009) with parameters “-gt figure0.8 -st 0.001 -cons 60”. The ML phylogenetic tree and trimmed CDS were used to estimate the divergence using the MCMCTree function in the PAML package (Yang 2007) with the approximate likelihood method, allowing independent substitution rates and the JC69 model (chosen as Nascimento *et al.* (2017) suggested). We ran 6 million iterations and discarded the first third of them as burn-in. To check for convergence with the stationary distribution, each analysis was run in duplicate and the results were compared between runs. The root age of the tree was calibrated to 48–52 Ma following Chen *et al.* (2019) and the crown age of the *Chamaetia-Vetrix* clade (here including *S. brachista*, *S. purpurea, S. suchowensis*, and *S. viminalis*) was calibrated to 23–25 Ma according to Wu *et al.* (2015).

We performed collinearity analysis of *P. trichocarpa* and the five willows, and self-comparison of each species, using MCScanX with the default parameters (Wang *et al.* 2012). KaKs_Calculator (Wang *et al.* 2010) was used to calculate *K*a (the number of substitutions per nonsynonymous site), *K*s (substitutions per synonymous site), and *K*a/*K*s values based on orthologous pairs, using the suggested Yang-Nielsen (YN) model by Zhang & Yu (2006).

# Supplementary Figures

## Figure S1 The bottom left part shows genome-wide Hi-C contact interactions of *Salix dunnii*, the upper right part shows the repeat sequences density of *Salix dunnii* genome of each chromosome. The black lines and block show the possible centromeric region of chromosome 7 based on the joint map.


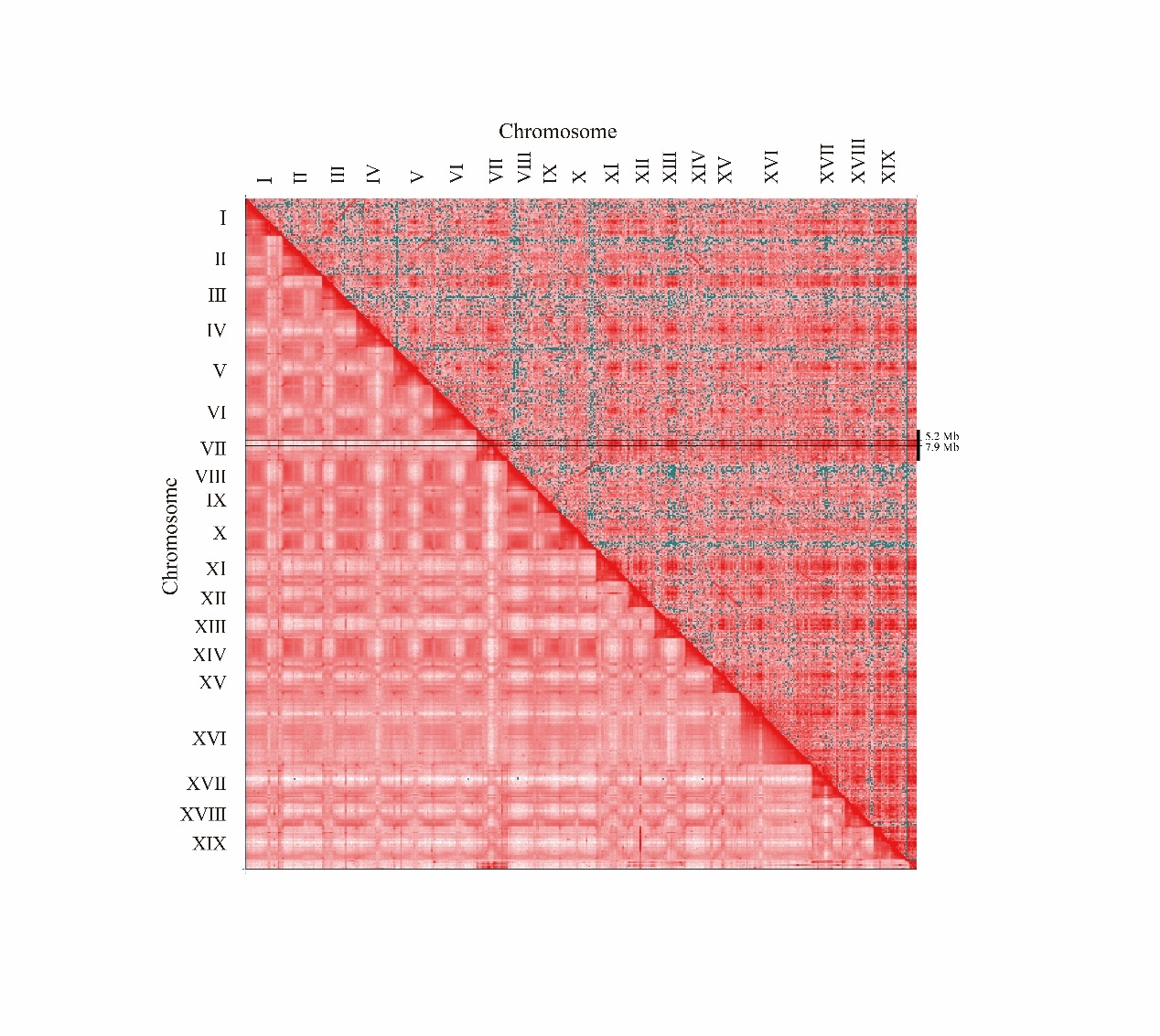


## Figure S2 Flow cytometry histograms of FAFU-HL-1 of *Salix dunnii* (a) and the external diploid standard *S. integra* (b).


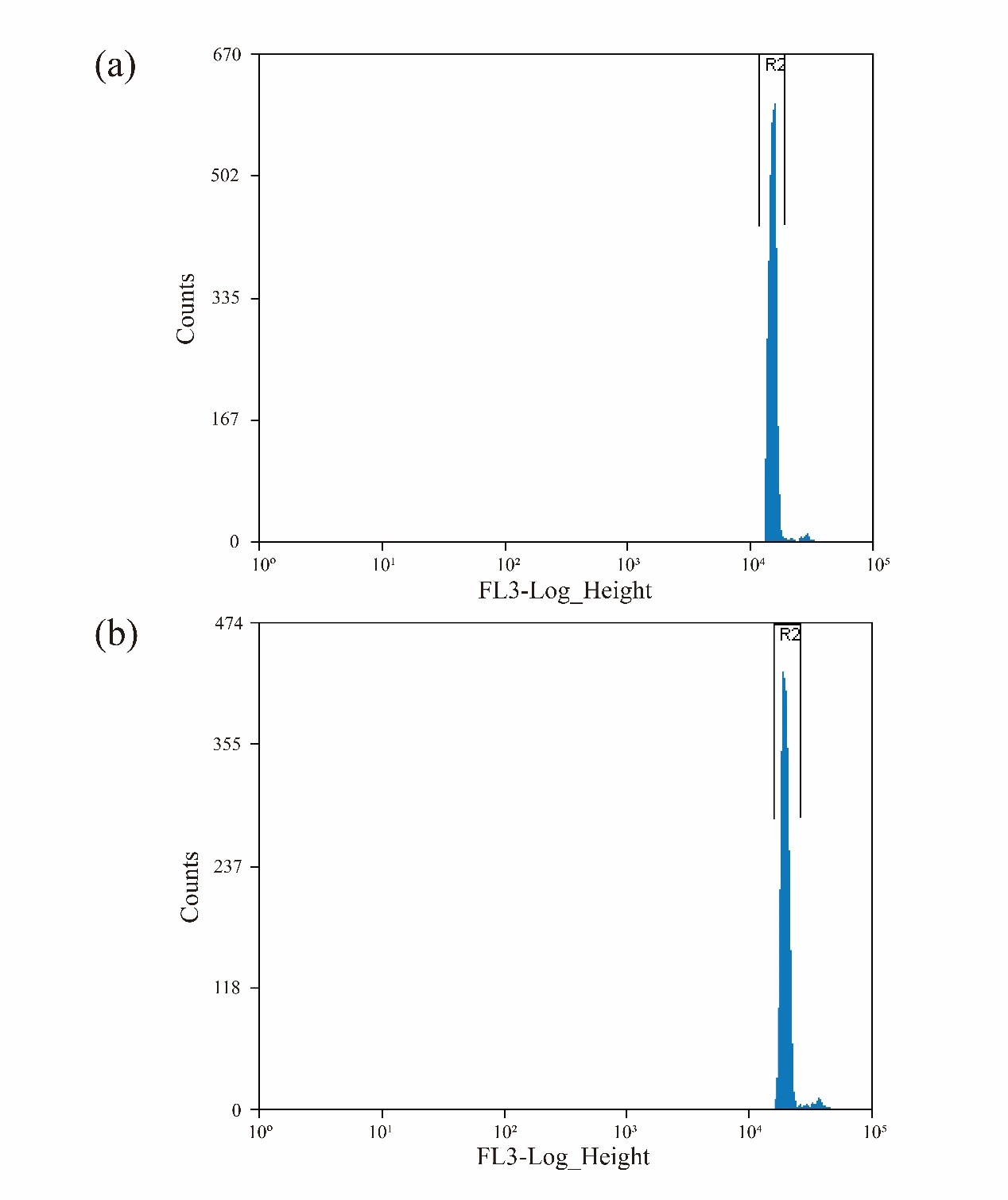


## Figure S3 The 17-mer distribution of Illumina PCR-free short-read data. The x-axis shows *k*-mer abundance; the y-axis shows the number of *k*-mer. The solid line represents *Ks* distribution. The dotted red line represents theoretical values.


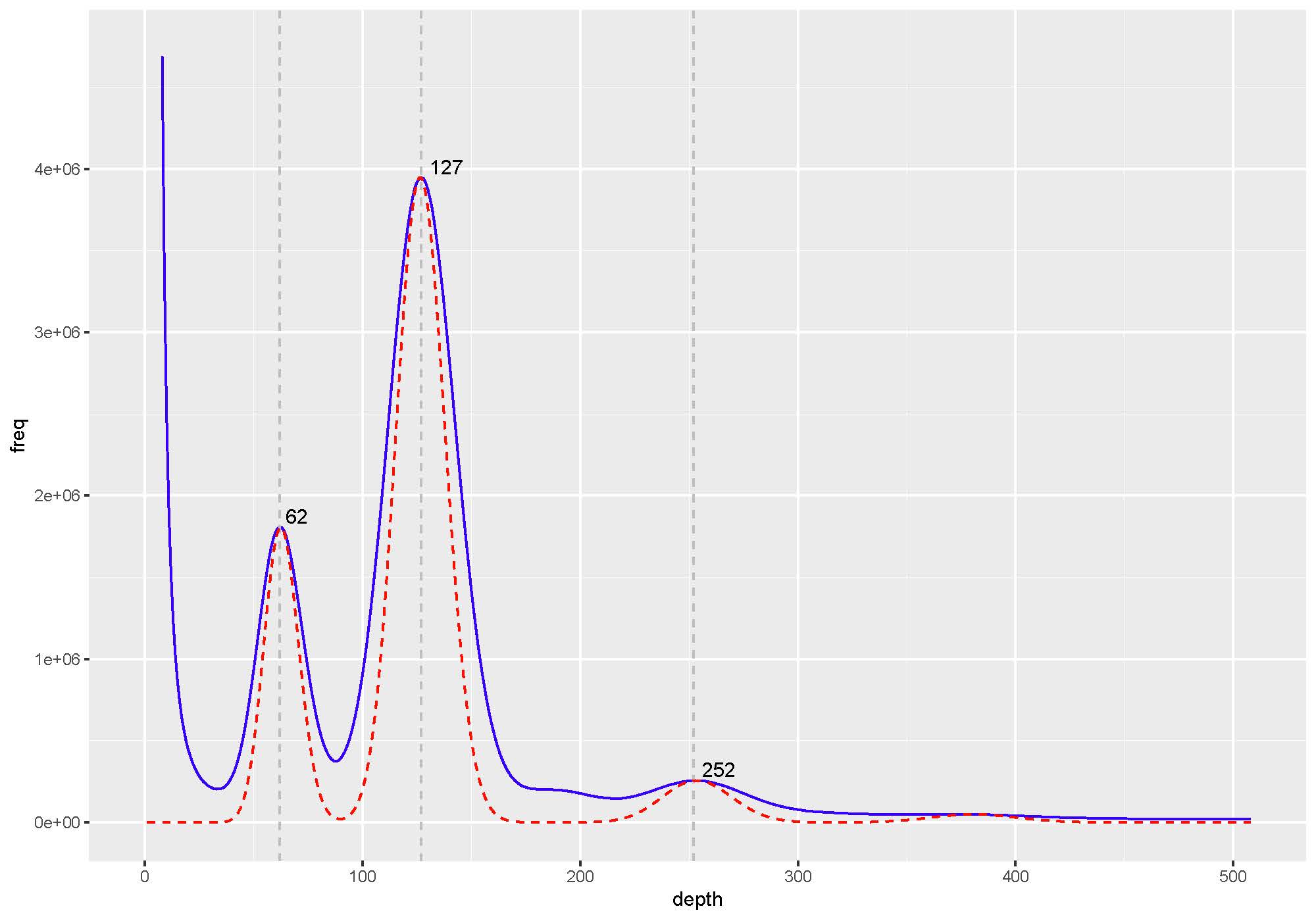


## Figure S4 Hi-C interaction heatmap of *Salix dunniii* pseudo-chromosome assembly. The resolution used to estimate the interaction strength of each bin is 100 kb.


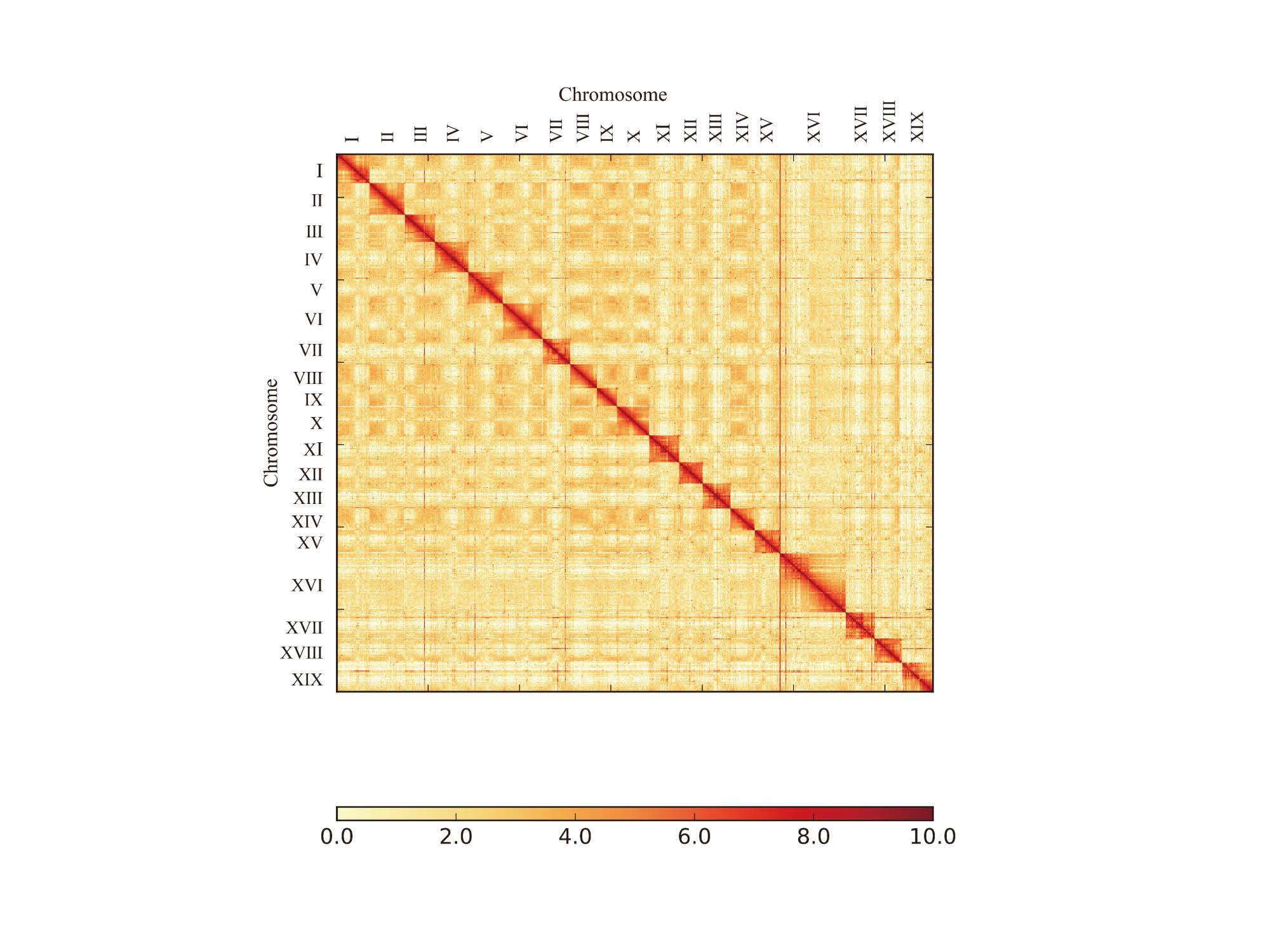


## Figure S5 Mitochondrial genome of *Salix dunnii*. Genomic features are shown facing outward (positive strand) and inward (negative strand) of the *Salix dunnii* mitochondrial genome represented as a circular molecule. The colour key shows the functional class of the mitochondrial genes. The GC content is represented in the innermost circle.


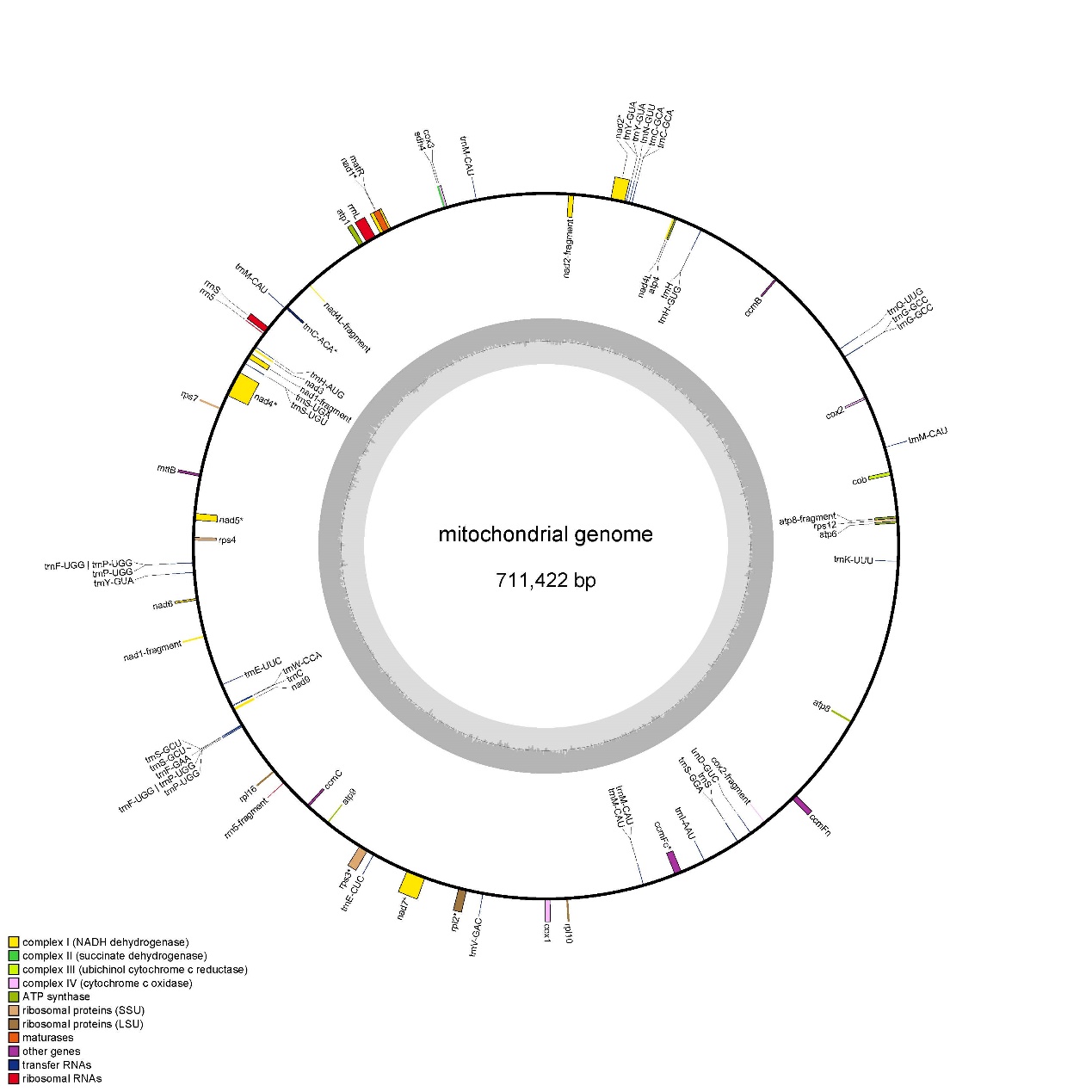


## Figure S6 Plastid genome of *Salix dunnii*. Genomic features are shown facing outward (positive strand) and inward (negative strand) of the circular *S. dunnii* plastid genome. The colour key shows the functional class of the plastid genes. The GC content is represented in the innermost circle with the inverted repeat (IR) and single copy (SC) regions indicated.


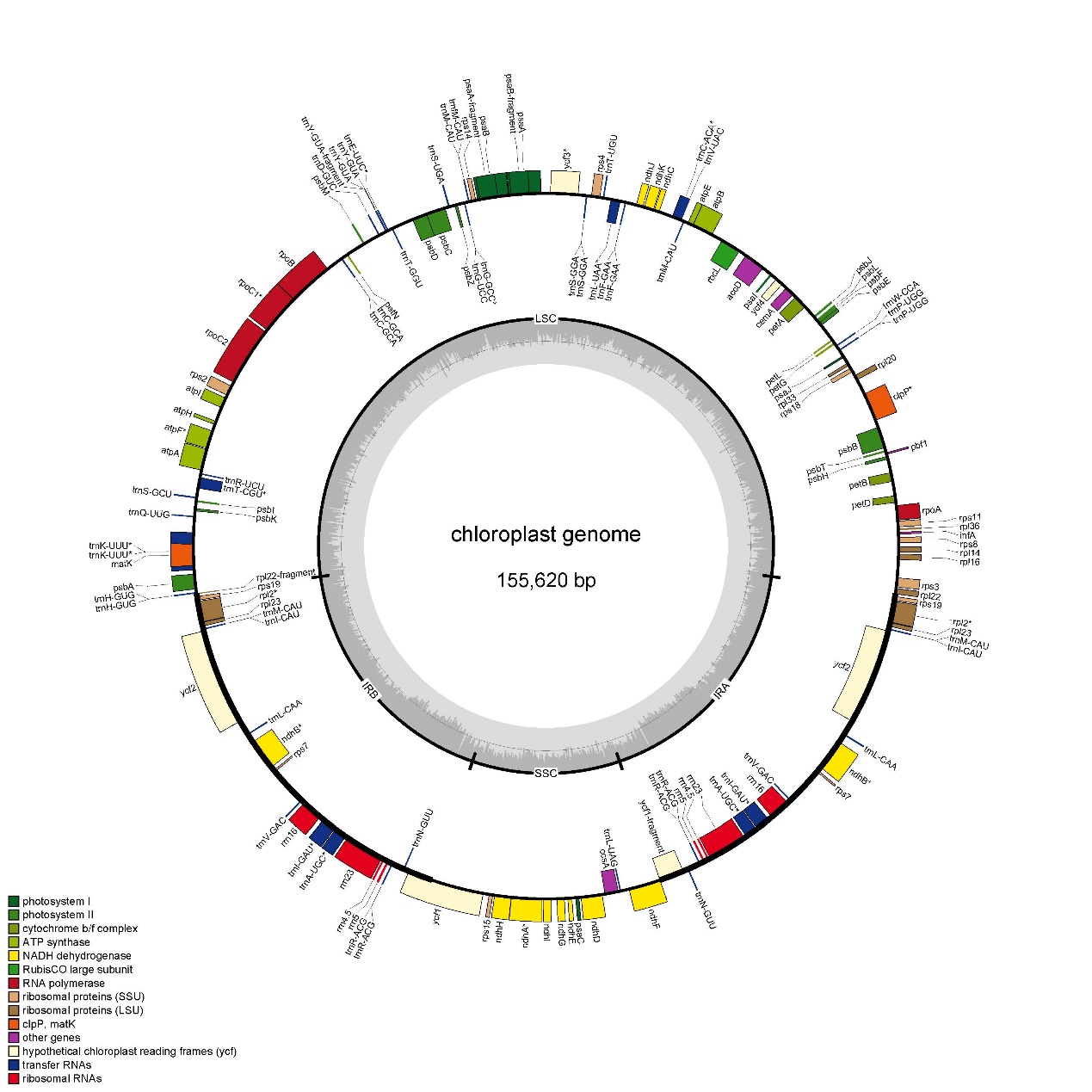


## Figure S7 Insertion time of LTR-RTs (long terminal repeat-retrotransposons) in the genome *Salix dunnii*.


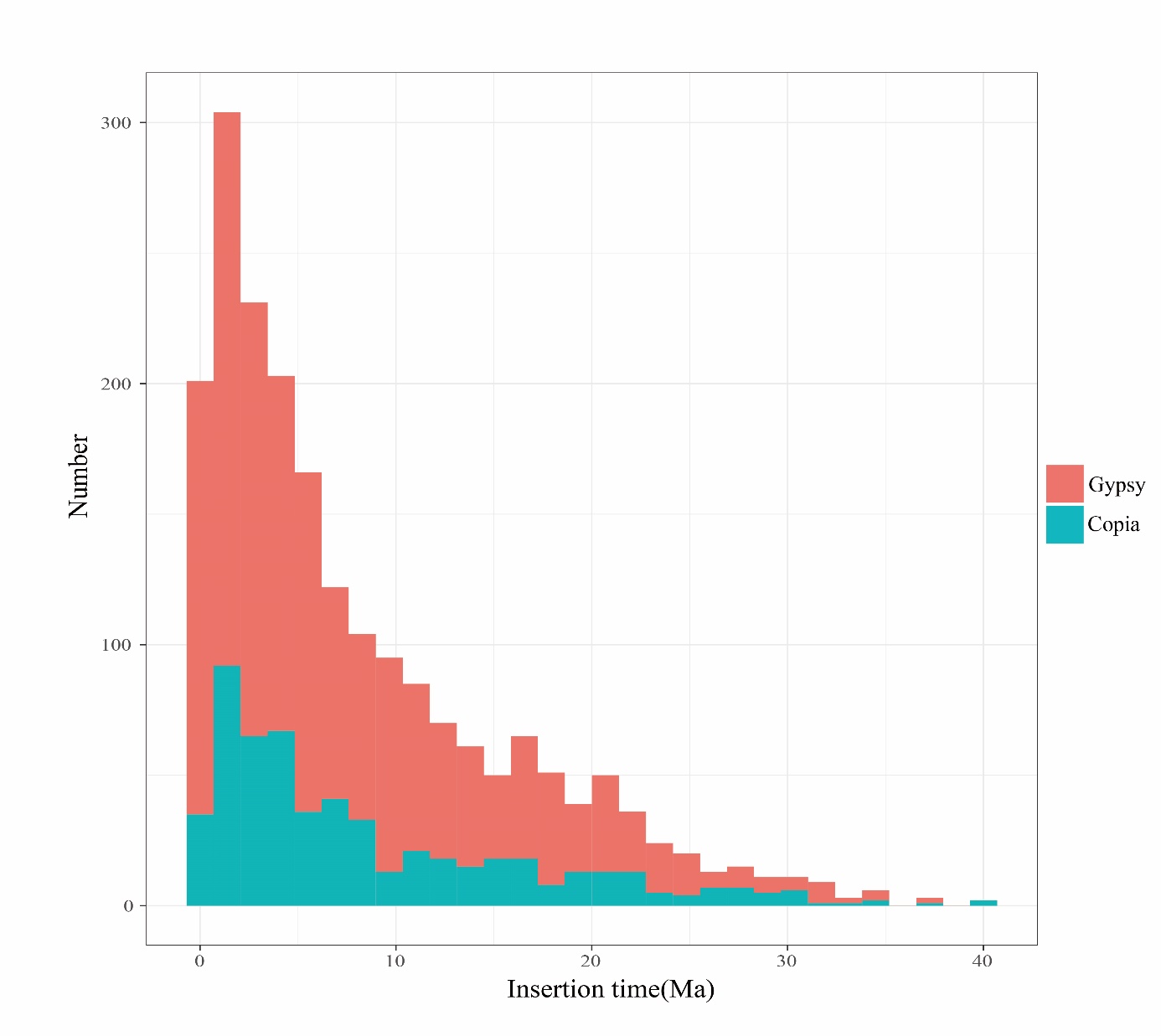


## Figure S8 Proliferation history of different superfamilies of the *Copia* class of LTR-RTs in the *Salix dunnii* genome*.*


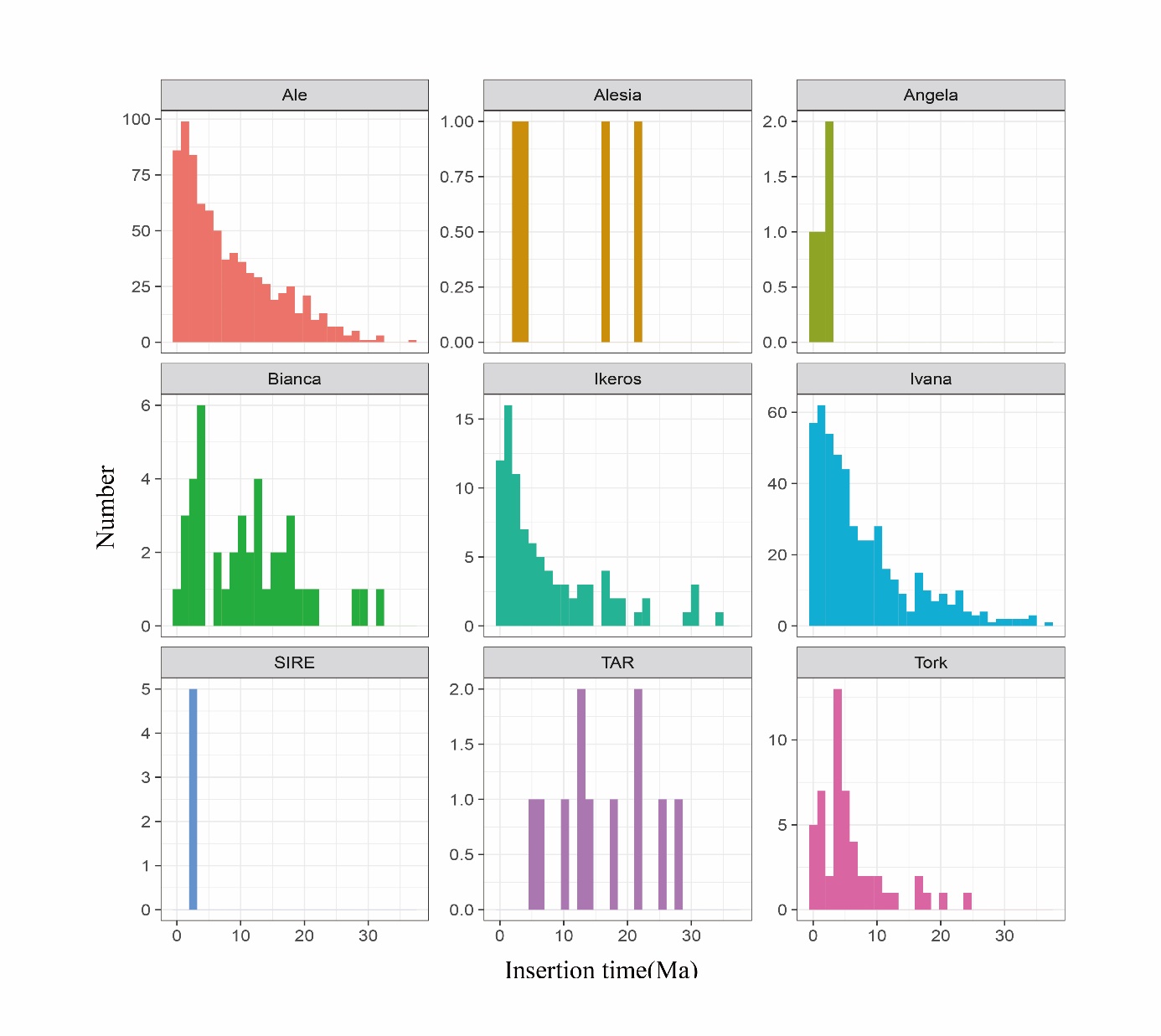


## Figure S9 Proliferation history of different superfamilies of the *Gypsy* class of LTR-RTs in the *Salix dunnii* genome*.*


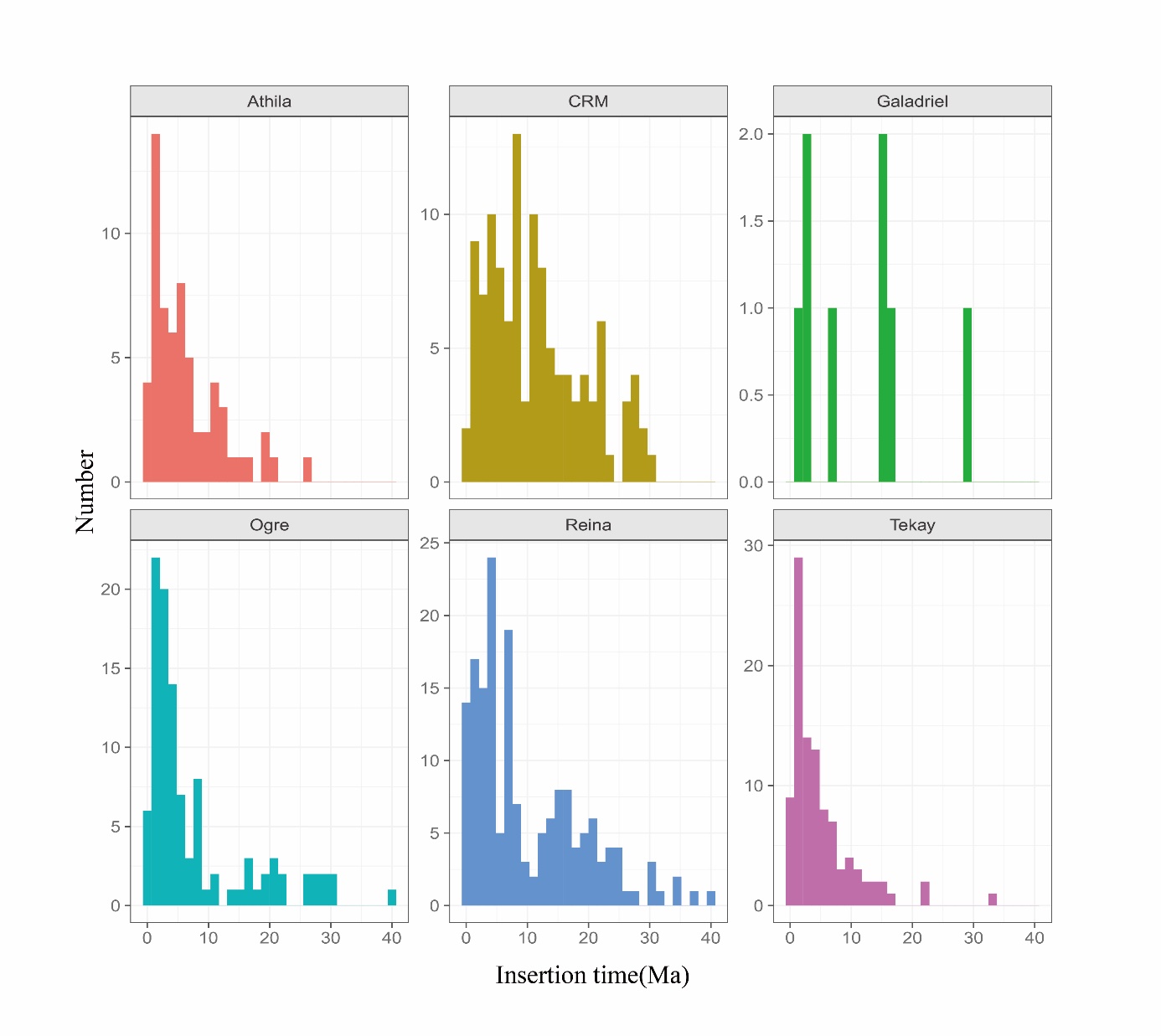


## Figure S10 *K*s values distribution for homologous in *Salix brachista*, *S. dunnii*, *S. purpurea*, *S. viminalis*, *S. suchowensis*, and *Populus trichocarpa*. (a) five *Salix* species pairs and *P. trichocarpa*; (b) between *P. trichocarpa* and five *Salix* species. *Salix* species and *Populus* species shared the same WGD event with *K*s value about 0.33 and 0.25, respectively. The peaks of divergence of *Populus* and *Salix* is around the *K*s value of 0.14.


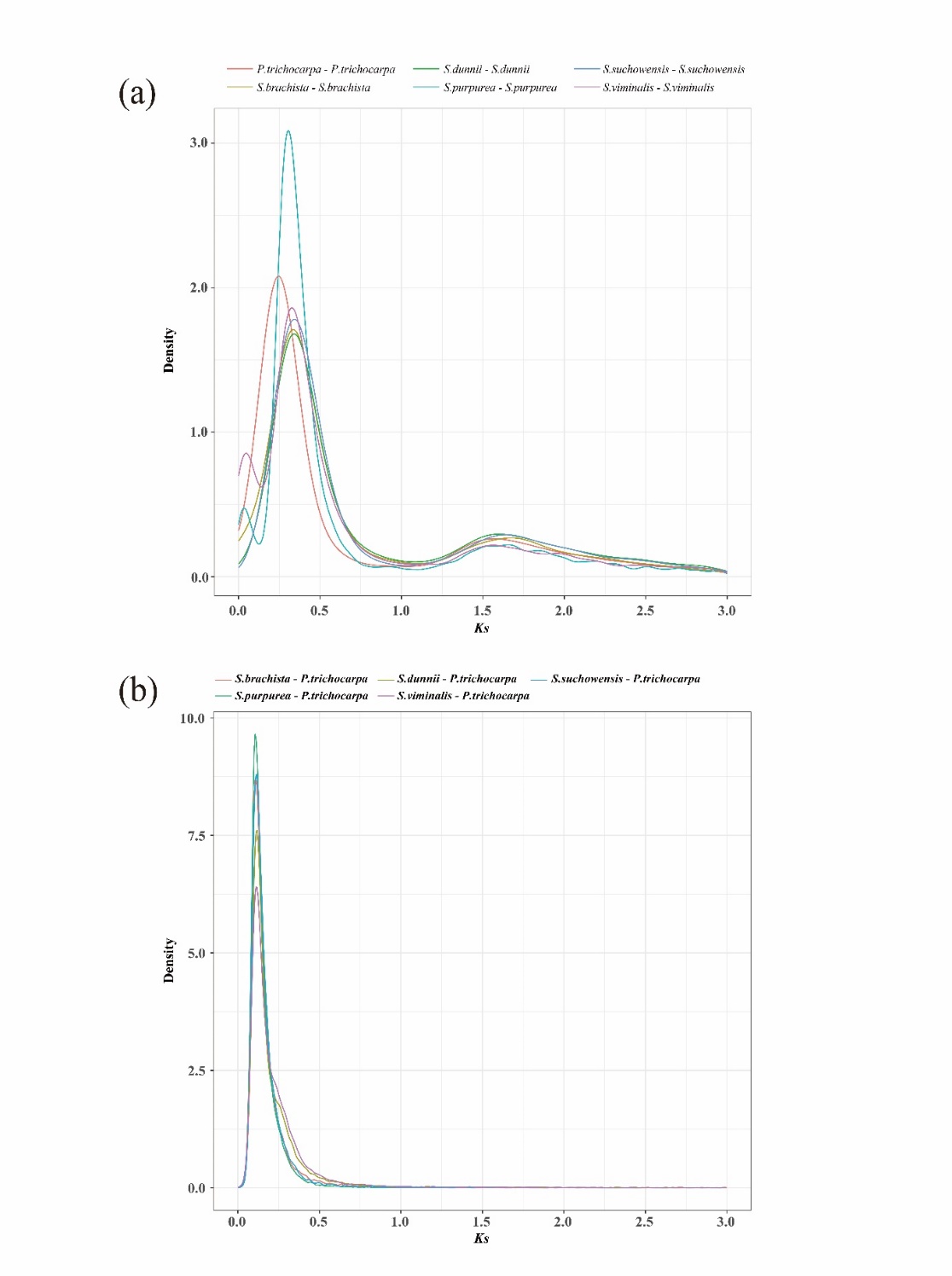


## Figure S11 Syntenic dot plot of the self-comparison of *Salix dunnii*.


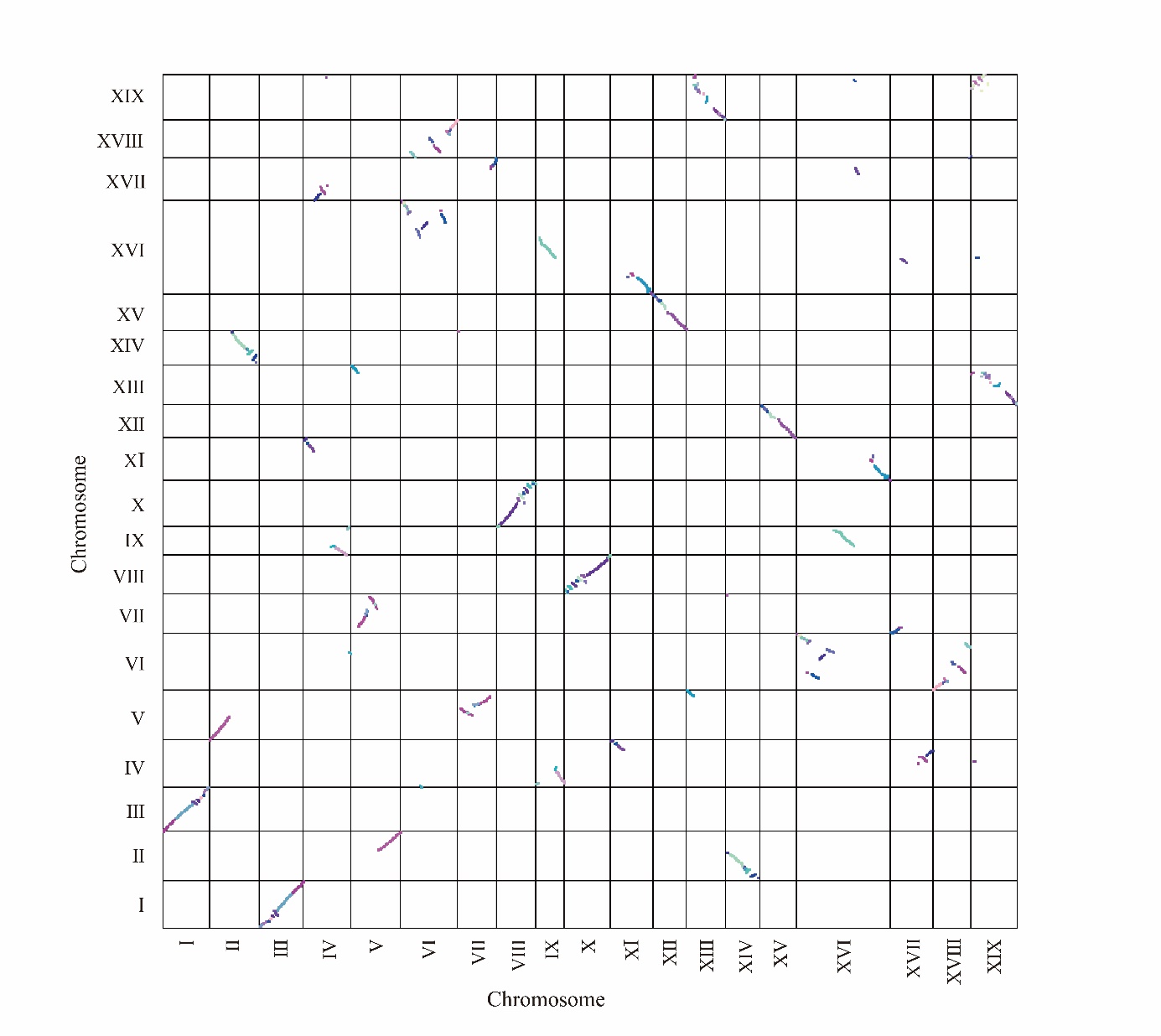


## Figure S12 Quantile–Quantile (Q–Q) plots of observed and expected GWAS P-values. Red dotted line indicates X = Y and blue shading the 95% confidence interval around the expectation of X = Y, that is that allele frequencies and sex are independent.


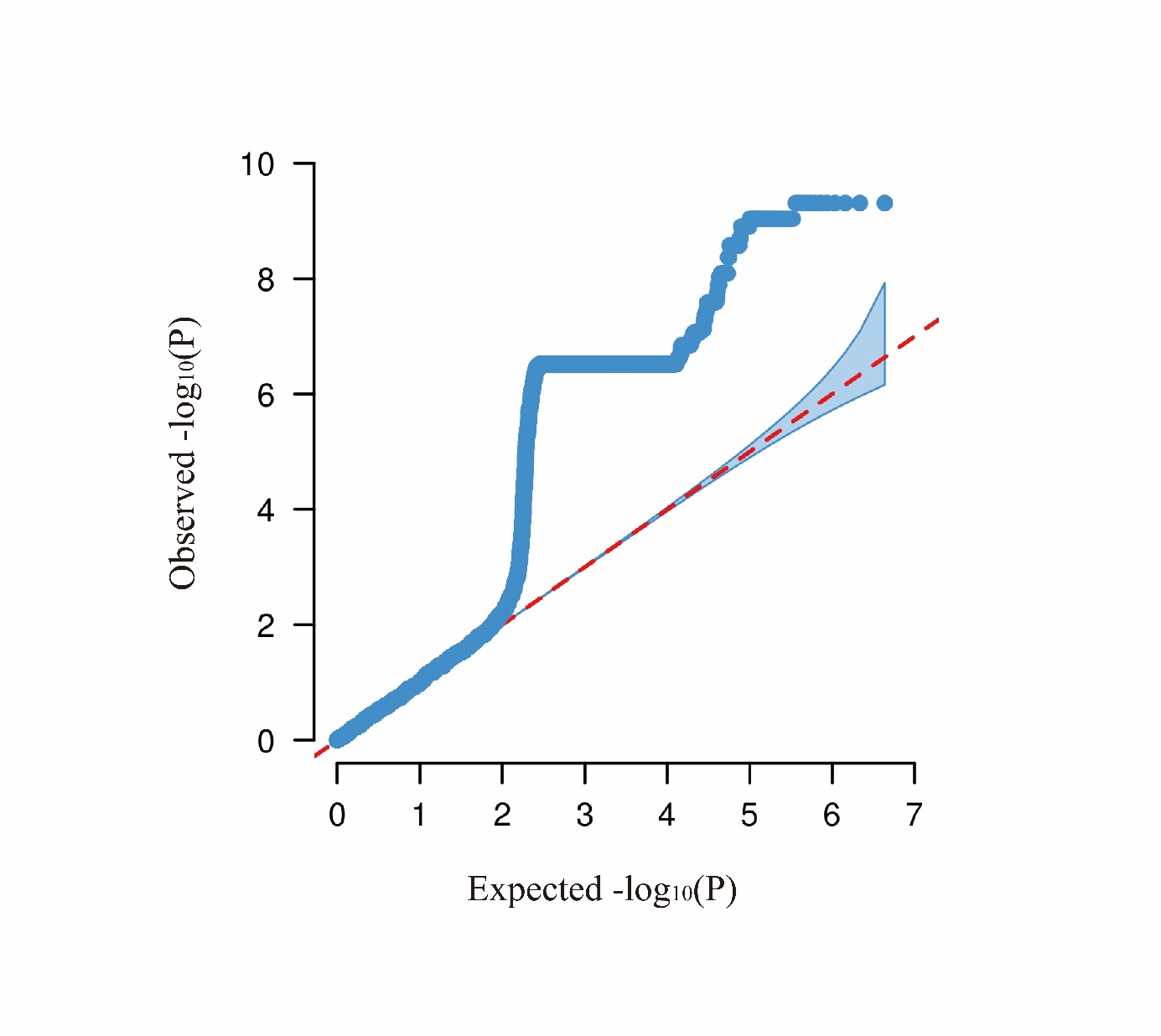


## Figure S13 Chromosome quotients (CQ) of each 50 kb nonoverlapping window of whole genome of *Salix dunnii*.


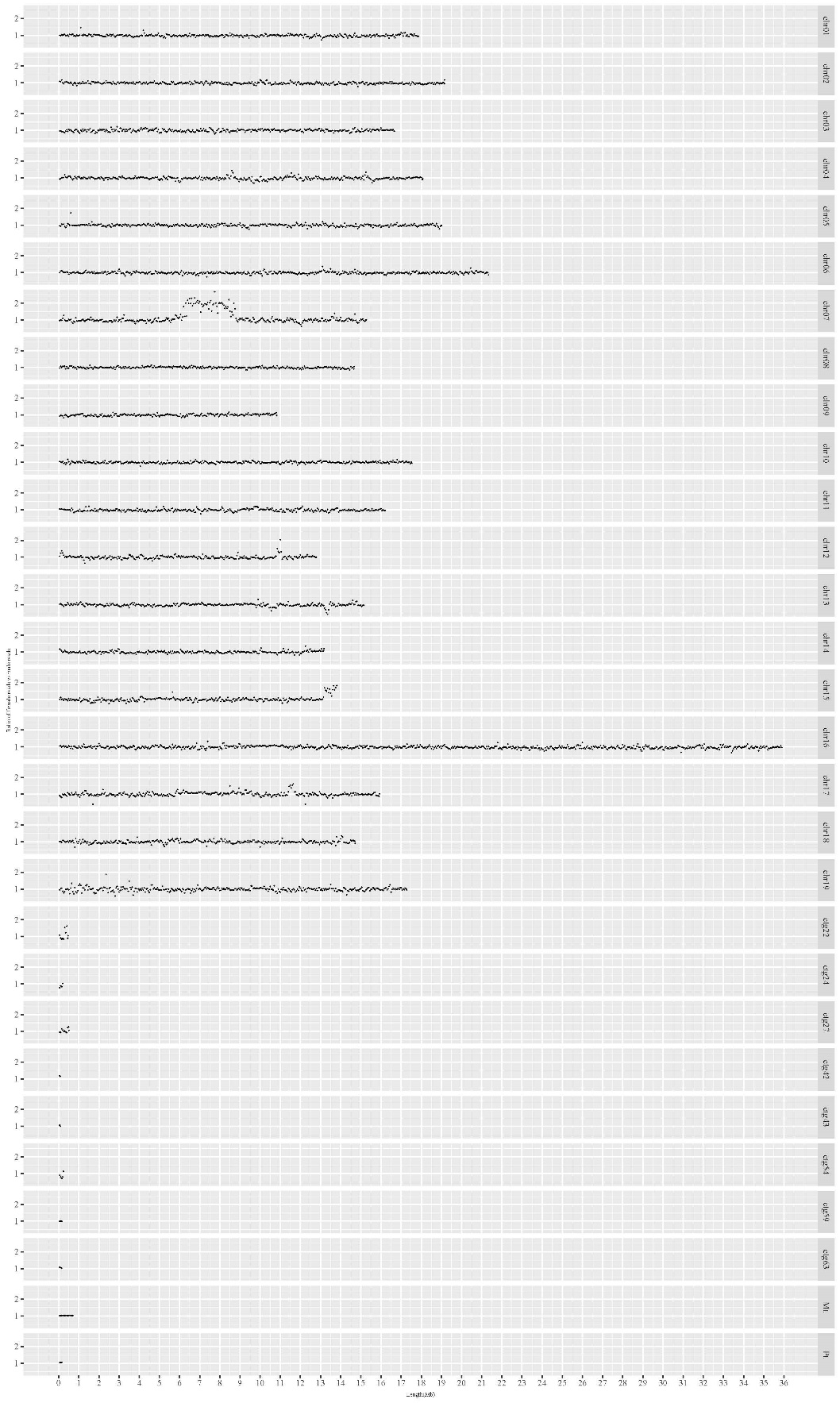


## Figure S14 Linkage disequilibrium pattern of each chromosome of *Salix dunnii* based on 20 female individuals.


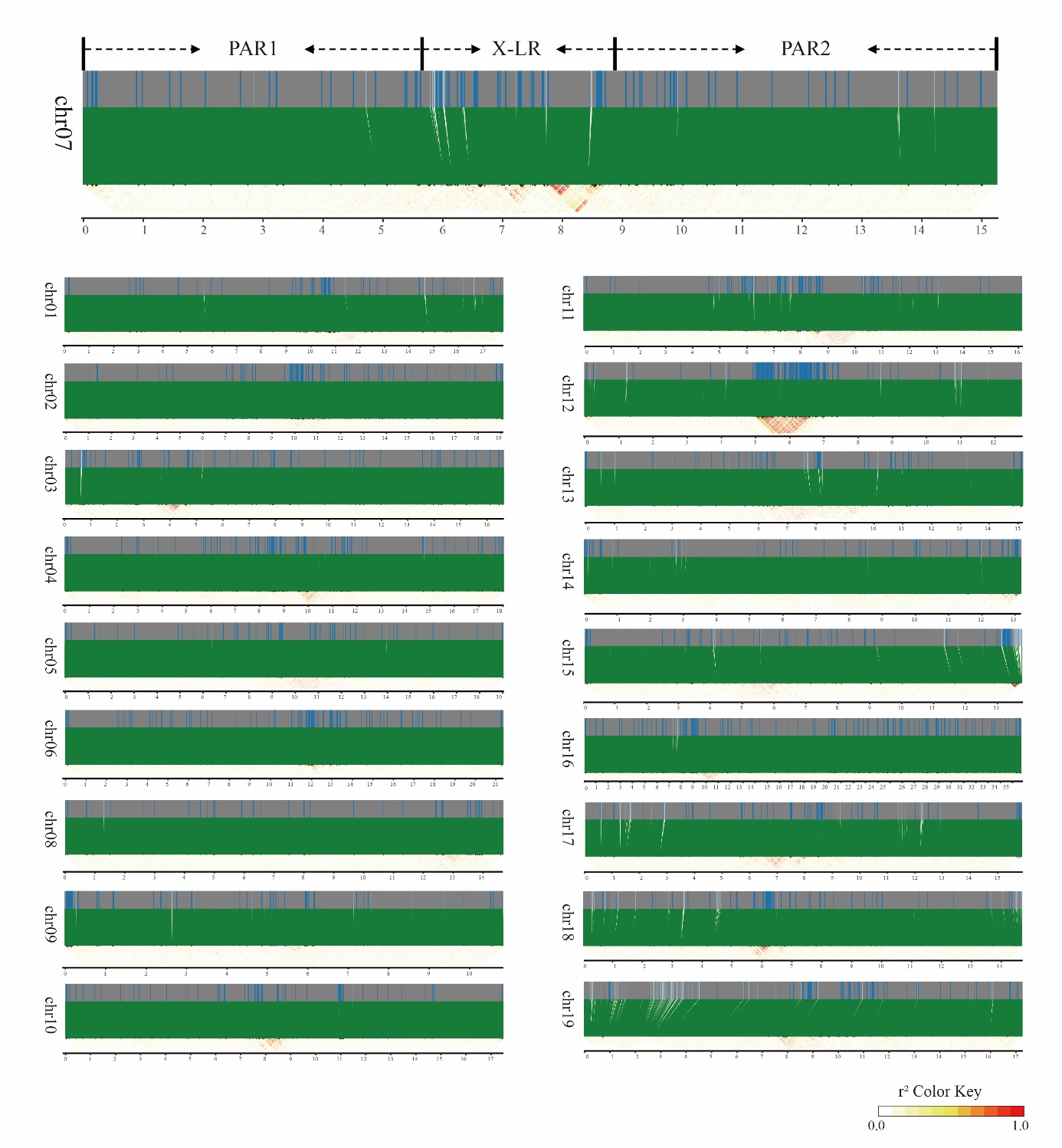


## Figure S15 Genome-wide plot of *F*_ST_-values of *Salix dunnii* calculated at 100 kb windows and 5 kb steps.


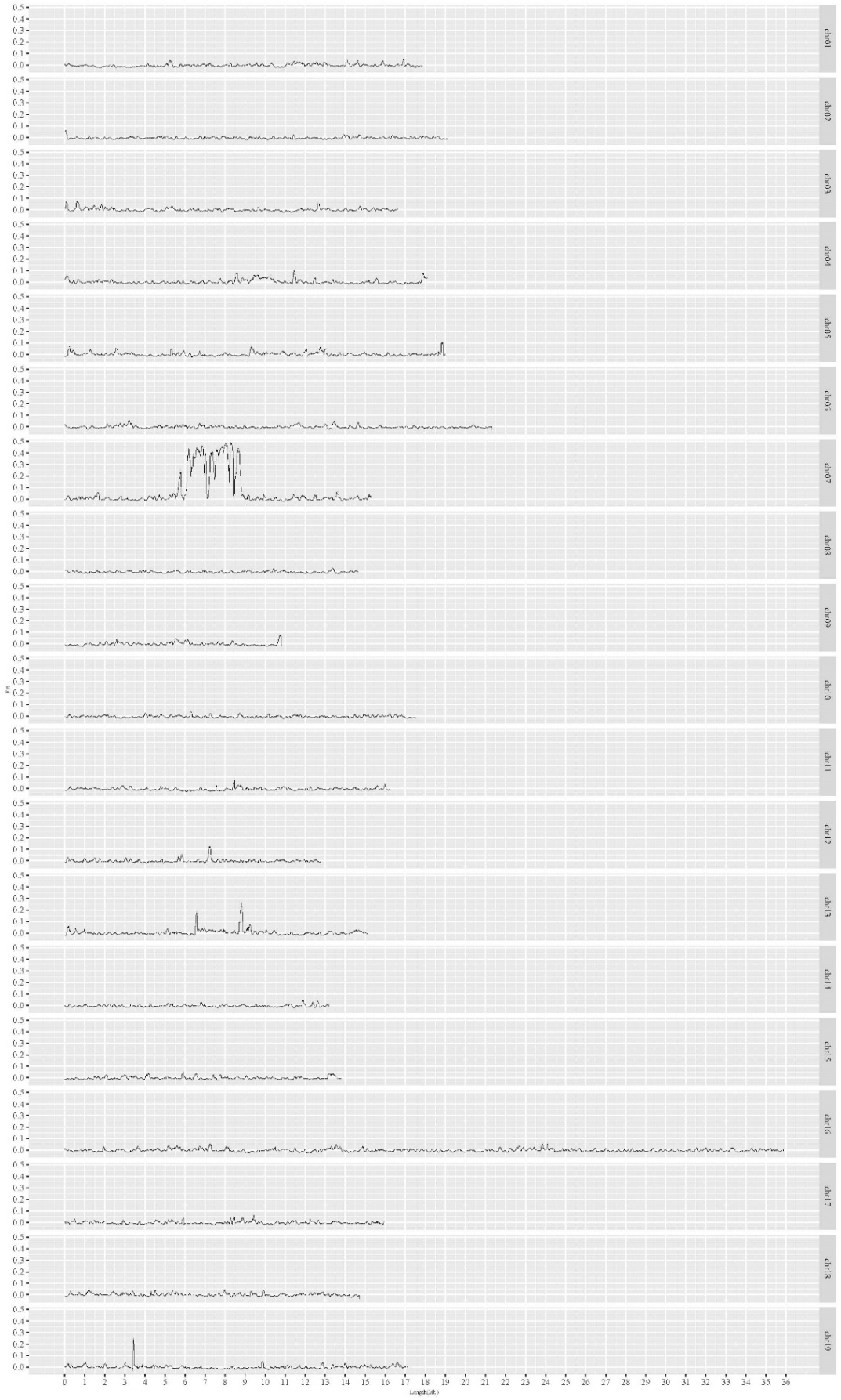


## Figure S16 Patterns of linkage disequilibrium decay in the whole genome of *Salix dunnii* (a) and in the X-linked region (b). LD is expressed as the squared allele frequency correlation (r^2^) between two sites whose distances apart are indicated on the X-axis.


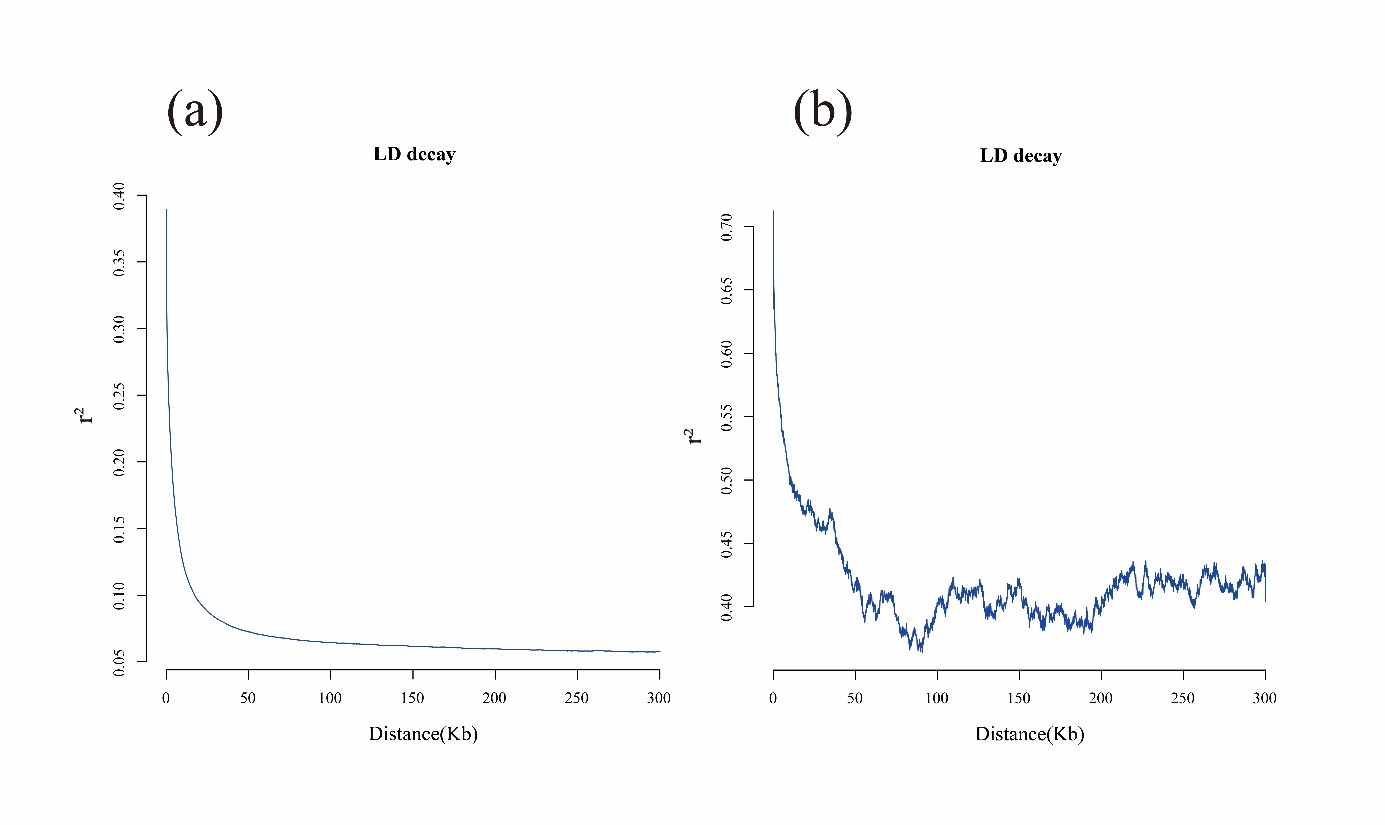


## Figure S17 Comparing *K*a and *K*s values of *S. dunnii-P. trichocarpa* homologous pairs between the chromosome 7 X-linked region, the two PARs, and autosomes. a, *K*a; b, *K*s; 990 homologous pairs (excluded 27 homologous pairs with *K*a or *K*s greater than 1) for chromosome 7, and 1846 for autosome (chromosome 6, excluded 51 homologous pairs with *K*a or *K*s greater than 1). c, *K*a; d, *K*s; 1017 homologous pairs for chromosome 7, and 1897 homologous pairs for autosome. The Wilcoxon rank sum test was used to detect the significant difference (*p* < 0.05). Red lines indicate median of *K*a and *K*s of autosome to make the differences easy to see.


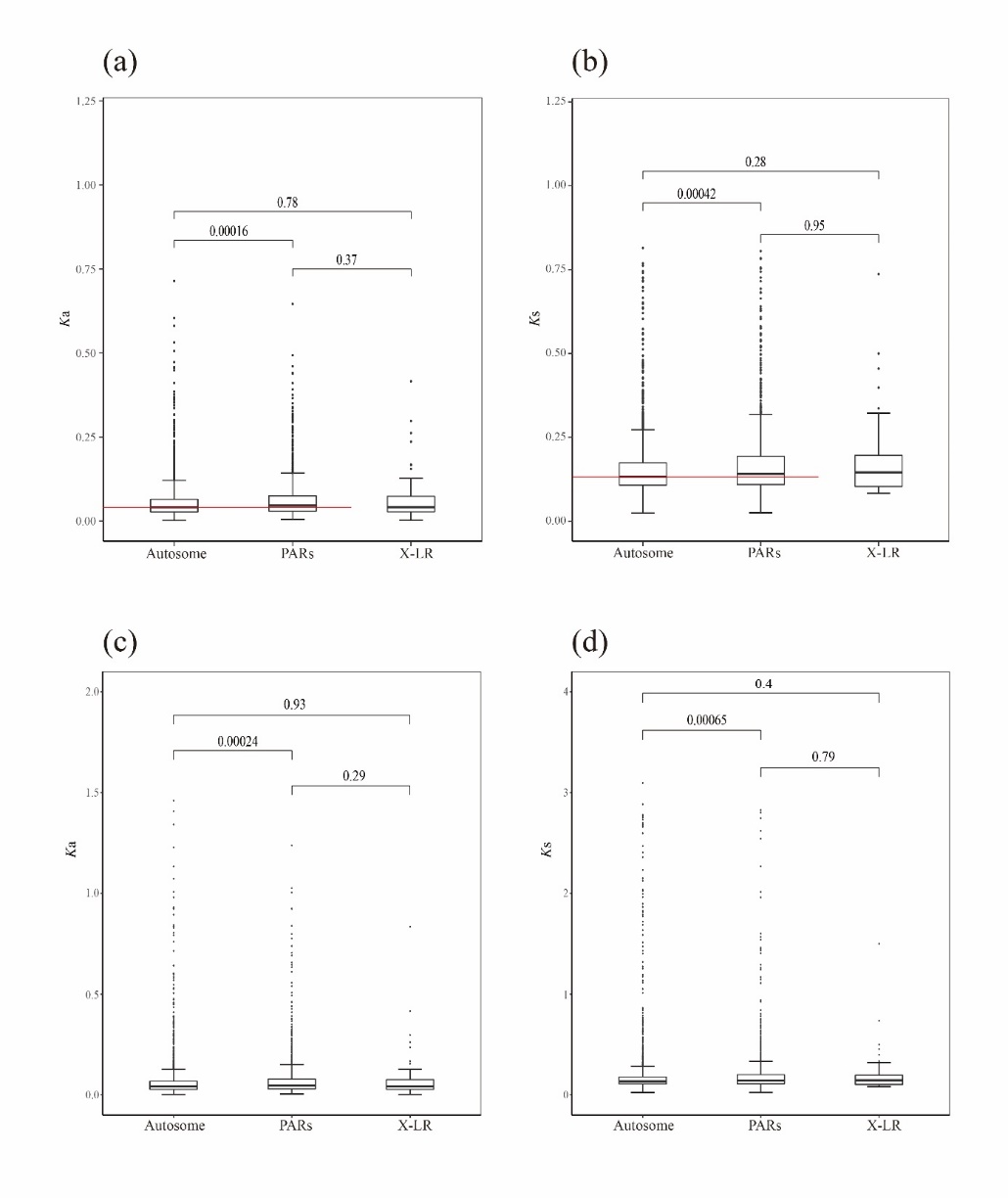


## Figure S18 Comparing *K*a and *K*s values of *S*. *dunnii-S. purpurea* homologous pairs between the chromosome 7 X-linked region, the two PARs, and autosomes. a, *K*a; b, *K*s; 965 homologous pairs (excluded 25 homologous pairs with *K*a or *K*s greater than 1) for chromosome 7, and 1808 for autosome (chromosome 6, excluded 44 homologous pairs with *K*a or *K*s greater than 1). c, *K*a; d, *K*s; 992 homologous pairs for chromosome 7, and 1852 homologous pairs for autosome*.* The Wilcoxon rank sum test was used to detect the significant difference (*p* < 0.05). Red lines indicate median of *K*a and *K*s of autosome to make the differences easy to see.


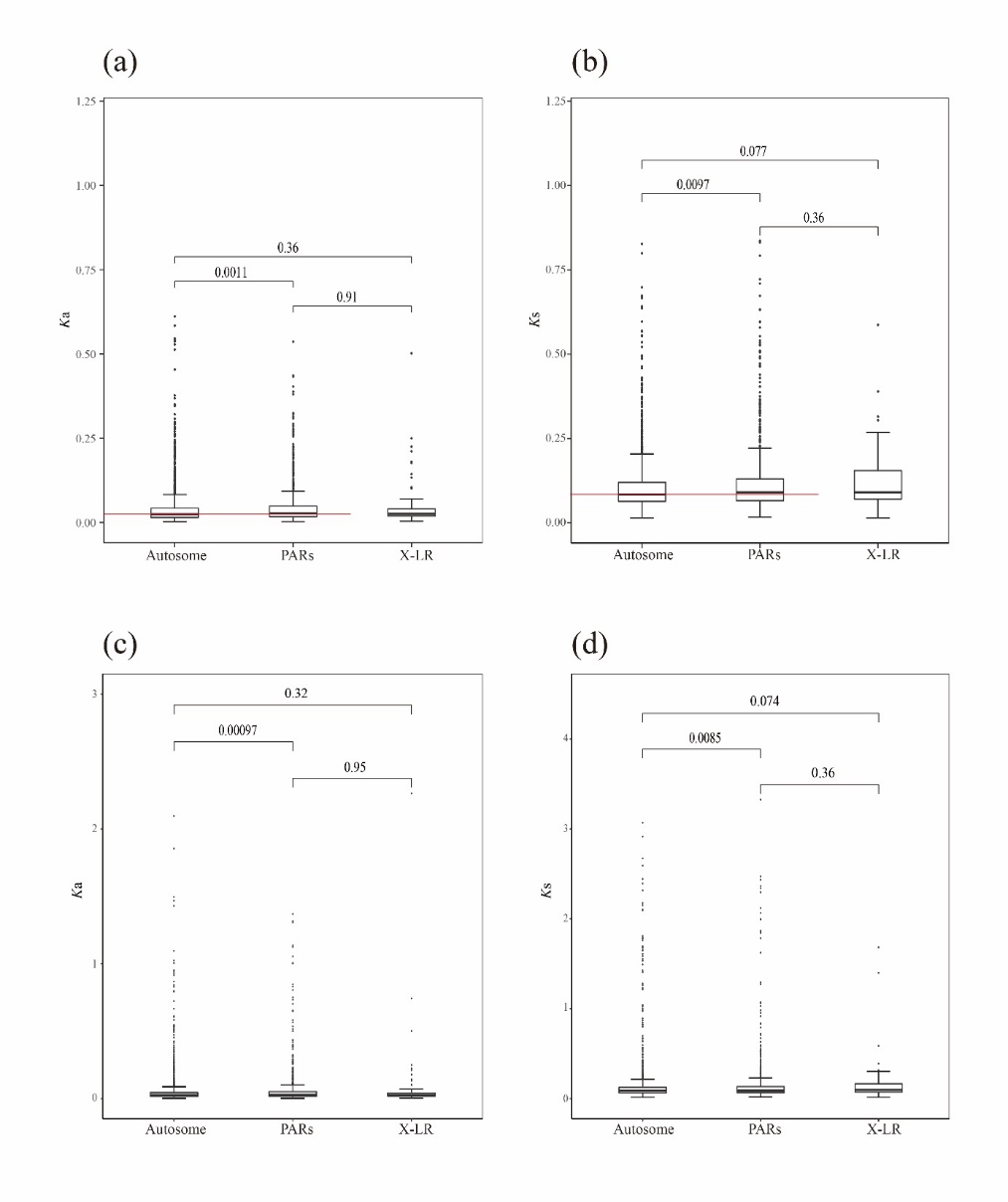


## Figure S19 Comparing *K*a/*K*s ratios between genes of the chromosome 7 X-linked region, the two PARs, and autosomes. a, *S. dunnii-P. trichocarpa* homologous pairs. b, *S*. *dunnii-S. purpurea* homologous pairs*.* The Wilcoxon rank sum test was used to detect the significant difference (*p* < 0.05).


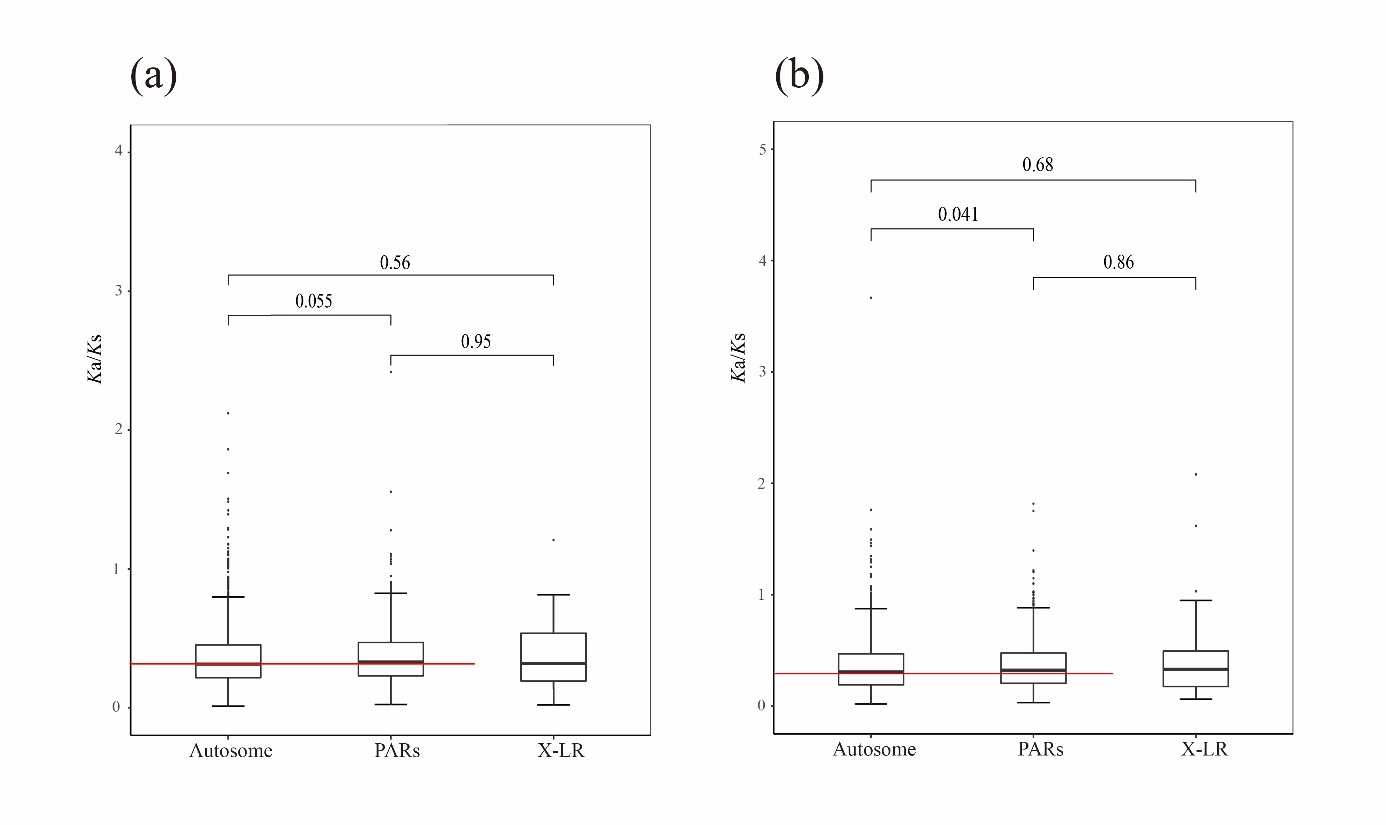


## Figure S20 Venn diagram comparing differential sex-biased expression genes in catkins and leaves.


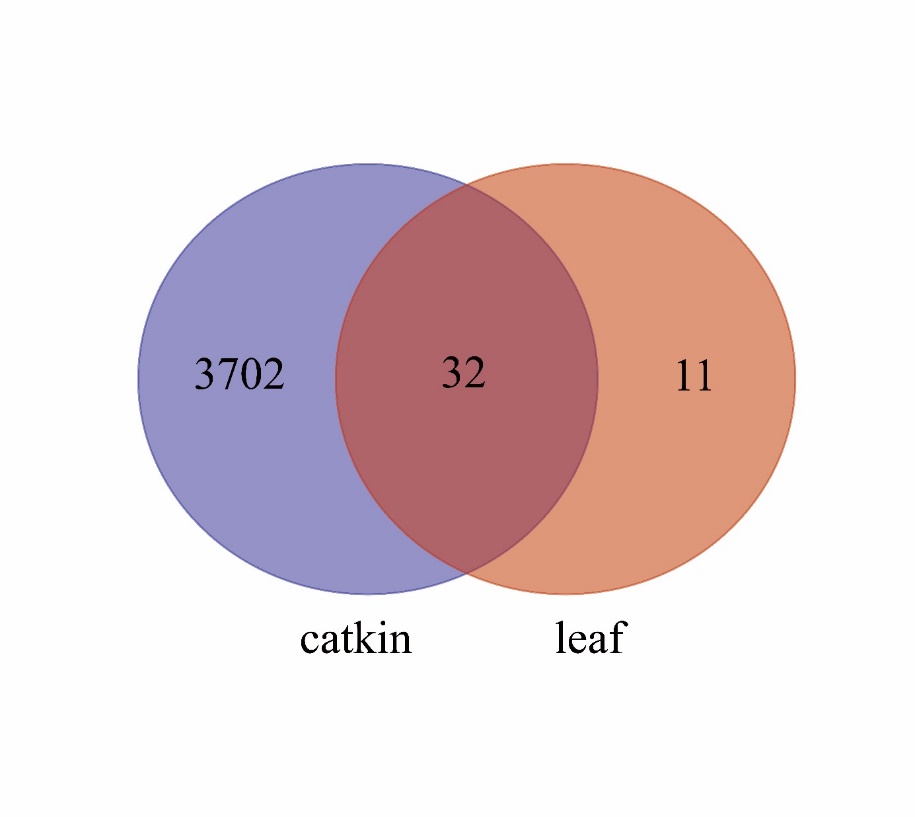


# Supplementary Tables (Independent xlsx file named as Table S1-S24)

## Table S1 Details of plant materials used in this study

## Table S2 Assembly statistics of different methods

## Table S3 Genome datasets used in the paper

## Table S4 Length statistics of the final reference genome of *Salix dunnii*

## Table S5 Statistics of the Oxford Nanopore Technologies (ONT) datasets

## Table S6 Details of DNA-seq and RNA-seq datasets used for assembly and annotation

## Table S7 Summary of repeat content of the genome of *Salix dunnii*

## Table S8 The statistics for full-length long terminal repeat-retrotransposons (LTR-RTs) of *Salix dunnii* genome

## Table S9 Distribution of RNAs on each regions of the genome of *Salix dunnii*

## Table S10 Statistics of RNAs of the genome of *Salix dunnii*

## Table S11 Functional annotation of the predicted genes of *Salix dunnii*

## Table S12 Transcription factor genes from 58 gene families of *Salix dunnii*

## Table S13 Summary of transcription factor genes of *Salix dunnii*

## Table S14 Statistics of quality control results of whole genome resequencing datasets

## Table S15 Summary of mapping results of 38 samples of *Salix dunnii*

## Table S16 Statistics of significantly sex associated SNPs in the female *Salix dunnii* genome regions

## Table S17 Statistics of heterozygosity analysis of the 101 sex associated SNPs

## Table S18 Genes in the X-linked region of *Salix dunnii*

## Table S19 Pseudogenes on chromosome 7 of *Salix dunnii*

## Table S20 Comparation of pseudogenes and genes on *Salix dunnii* genome

## Table S21 Homologous copies of ARR17 on the whole female genome of *Salix dunnii* searched by tblastn

## Table S22 Transcriptome data quality control and mapping results

## Table S23 The numbers of biased genes expression in catkins and leaves

## Table S24 Statistics of genome size, genes, and sex determination systems of the five willows with assembled genomes

# Softwares

JELLYFISH v2.2.10

findGSE v1.94

GenomeScope v1.0

SMARTdenovo v 1.0

wtdbg2 2.1

Canu v1.8

Pilon v1.22

fastp v0.19.5

Juicer 1.5.6

Juicebox 180922

Minimap2 2.17-r941 https://github.com/lh3/minimap2

LR_Gapcloser v1.1

NextPolish v1.2.4

RepeatMasker v4.0.7

LTRharvest 1.5.8

MAFFT v7.221

PASA 2.3.3 http://pasapipeline.github.io/

AUGUSTUS v3.2.3

SNAP version 2006-07-28 https://github.com/KorfLab/SNAP

MAKER v2.31.9

tRNAScan-SE 2.0.5

RNAMMER 1.2

BLAT v.36

InterProScan 5.27-66.0

BLAST v2.2.28+ and BLAST 2.9.0+

OrthoFinder2 v2.3.1

IQ-TREE v1.6.7

trimAl v1.4

PAML v4.9h

MCScanX (<https://github.com/wyp1125/MCScanX>)

KaKs_Calculator 2.0

BWA v0.7.12

SAMtools v1.9

Sambamba v0.7.0

FreeBayes v1.3.1-16-g85d7bfc

VCFtools v0.1.15

PLINK v1.90b3.42

Stacks v2.53

Changepoint package 2.2.2

PopLDdecay v3.40

LDBlockShow 1.36

MCscan (Python version，JCVI utility libraries v1.0.6)

ParaAT 2.0

CLUSTAL 2.1

STAR v2.7.3a

featureCounts v1.6.3

DEseq2 v1.30.0

HiSat2 v2.1.0

StringTie v1.3.3b

Trinity v2.0.6

CD-HIT v4.6

# Reference

Cantarel, B. L., Korf, I., Robb, S. M., Parra, G., Ross, E., Moore, B., . . . Yandell, M. (2008). MAKER: an easy-to-use annotation pipeline designed for emerging model organism genomes. *Genome Res, 18*(1), 188–196.

Capella-Gutiérrez, S., Silla-Martinez, J. M., & Gabaldon, T. (2009). trimAl: a tool for automated alignment trimming in large-scale phylogenetic analyses. *Bioinformatics, 25*(15), 1972–1973.

Chen, J. H., Huang, Y., Brachi, B., Yun, Q. Z., Zhang, W., Lu, W., . . . Sun, H. (2019). Genome-wide analysis of Cushion willow provides insights into alpine plant divergence in a biodiversity hotspot. *Nat Commun*, 10(1), 5230.

Chen, S., Zhou, Y., Chen, Y., & Gu, J. (2018). fastp: an ultra-fast all-in-one FASTQ preprocessor. *Bioinformatics*, 34(17), i884–i890.

Doležel, J., Greilhuber, J., & Suda, J. (2007). Estimation of nuclear DNA content in plants using flow cytometry. *Nat Protoc, 2*(9), 2233–2244.

Emms, D. M., & Kelly, S. (2019). OrthoFinder: phylogenetic orthology inference for comparative genomics. *Genome Biol, 20*(1), 238.

Fu, L., Niu, B., Zhu, Z., Wu, S., & Li, W. (2012). CD-HIT: accelerated for clustering the next-generation sequencing data. *Bioinformatics, 28*(23), 3150–3152.

Haas, B. J., Delcher, A. L., Mount, S. M., Wortman, J. R., Smith, R. K., Hannick, L. I., . . . White, O. (2003). Improving the Arabidopsis genome annotation using maximal transcript alignment assemblies. *Nucleic Acids Res, 31*(19), 5654–5666.

Haas, B. J., Papanicolaou, A., Yassour, M., Grabherr, M., Blood, P. D., Bowden, J., . . . Dewey, C. (2013). De novo transcript sequence reconstruction from RNA-seq using the Trinity platform for reference generation and analysis. *Nat Protoc, 8*(8), 1494–1512.

He, L., Wagner, N. D., & Hörandl, E. (2021). Restriction-site associated DNA sequencing data reveal a radiation of willow species (Salix L., Salicaceae) in the Hengduan Mountains and adjacent areas. *J Syst Evol*, *59*(1), 44–57.

Jones, P., Binns, D., Chang, H. Y., Fraser, M., Li, W., McAnulla, C., . . . Hunter, S. (2014). InterProScan 5: genome-scale protein function classification. *Bioinformatics, 30*(9), 1236–1240.

Katoh, K., & Standley, D. M. (2013). MAFFT multiple sequence alignment software version 7: improvements in performance and usability. *Mol Biol Evol, 30*(4), 772–780.

Kent, W. J. (2002). BLAT--the BLAST-like alignment tool. *Genome Res, 12*(4), 656–664.

Kim, D., Langmead, B., & Salzberg, S. L. (2015). HISAT: a fast spliced aligner with low memory requirements. *Nat Methods, 12*(4), 357–360.

Korf, I. (2004). Gene finding in novel genomes. *BMC Bioinformatics, 5*, 59.

Lagesen, K., Hallin, P., R, D. E. A., Staerfeldt, H.-H., Rognes, T. R., & Ussery, D. W. (2007). RNAmmer: consistent and rapid annotation of ribosomal RNA genes. *Nucleic Acids Res, 35*(9), 3100–3108.

Lowe, T. M., & Eddy, S. R. (1997). tRNAscan-SE: a program for improved detection of transfer RNA genes in genomic sequence. *Nucleic Acids Res, 25*(5), 955–964.

Nascimento, F. F., Reis, M. D., & Yang, Z. (2017). A biologist's guide to Bayesian phylogenetic analysis. *Nat Ecol Evol, 1*(10), 1446–1454.

Nawrocki, E. P., Burge, S. W., Bateman, A., Daub, J., Eberhardt, R. Y., Eddy, S. R., . . . Finn, R. D. (2015). Rfam 12.0: updates to the RNA families database. *Nucleic Acids Res, 43*(Database issue), D130–137.

Nguyen, L. T., Schmidt, H. A., von Haeseler, A., & Minh, B. Q. (2015). IQ-TREE: a fast and effective stochastic algorithm for estimating maximum-likelihood phylogenies. *Mol Biol Evol, 32*(1), 268–274.

Pertea, M., Pertea, G. M., Antonescu, C. M., Chang, T. C., Mendell, J. T., & Salzberg, S. L. (2015). StringTie enables improved reconstruction of a transcriptome from RNA-seq reads. *Nat Biotechnol*, 33(3), 290–295.

Powell, S., Forslund, K., Szklarczyk, D., Trachana, K., Roth, A., Huerta-Cepas, J., . . . Bork, P. (2014). eggNOG v4.0: nested orthology inference across 3686 organisms. *Nucleic Acids Res, 42*(Database issue), D231–239.

Stanke, M., Diekhans, M., Baertsch, R., & Haussler, D. (2008). Using native and syntenically mapped cDNA alignments to improve de novo gene finding. *Bioinformatics, 24*(5), 637–644.

Wagner, N. D., He, L., & Horandl, E. (2020). Phylogenomic Relationships and Evolution of Polyploid Salix Species Revealed by RAD Sequencing Data. *Front Plant Sci, 11*, 1077.

Wang, D., Zhang, Y., Zhang, Z., Zhu, J., & Yu, J. (2010). KaKs_Calculator 2.0: a toolkit incorporating gamma-series methods and sliding window strategies. *Genom Proteom Bioinf, 8*(1), 77–80.

Wang, Y., Tang, H., Debarry, J. D., Tan, X., Li, J., Wang, X., . . . Paterson, A. H. (2012). MCScanX: a toolkit for detection and evolutionary analysis of gene synteny and collinearity. *Nucleic Acids Res, 40*(7), e49.

Wu, J., Nyman, T., Wang, D. C., Argus, G. W., Yang, Y. P., & Chen, J. H. (2015). Phylogeny of Salix subgenus Salix s.l. (Salicaceae): delimitation, biogeography, and reticulate evolution. *BMC Evol Biol, 15*, 31.

Yang, Z. (2007). PAML 4: Phylogenetic Analysis by Maximum Likelihood. *Mol Biol Evol, 24*(8), 1586–1591.

Zhang, Z., Carriero, N., Zheng, D., Karro, J., Harrison, P. M., & Gerstein, M. (2006). PseudoPipe: an automated pseudogene identification pipeline. *Bioinformatics, 22*(12), 1437–1439.

Zhang, Z., & Yu, J. (2006). Evaluation of six methods for estimating synonymous and nonsynonymous substitution rates. *Genom Proteom Bioinf, 4*(3), 173–181.
